# Transcriptome analysis of AAV-induced retinopathy models expressing human VEGF, TNF-α, and IL-6 in murine eyes

**DOI:** 10.1038/s41598-022-23065-4

**Published:** 2022-11-12

**Authors:** Kolja Becker, Carina M. Weigelt, Holger Fuchs, Coralie Viollet, Werner Rust, Hannah Wyatt, Jochen Huber, Thorsten Lamla, Francesc Fernandez-Albert, Eric Simon, Nina Zippel, Remko A. Bakker, Holger Klein, Norbert H. Redemann

**Affiliations:** 1 Global Computational Biology & Digital Sciences, Boehringer Ingelheim Pharma GmbH & Co. KG, Biberach an der Riß, Germany; 2 Cardiometabolic Diseases Research, Boehringer Ingelheim Pharma GmbH & Co. KG, Biberach an der Riß, Germany; 3 Drug Discovery Sciences, Boehringer Ingelheim Pharma GmbH & Co. KG, Biberach an der Riß, Germany; 4 Clinical Development & Operations Corporate, Boehringer Ingelheim Pharma GmbH & Co. KG, Biberach an der Riß, Germany

**Keywords:** Transcriptomics, Visual system, Animal disease models, Gene expression profiling, Retinal diseases, Computational biology and bioinformatics

## Abstract

Retinopathies are multifactorial diseases with complex pathologies that eventually lead to vision loss. Animal models facilitate the understanding of the pathophysiology and identification of novel treatment options. However, each animal model reflects only specific disease aspects and understanding of the specific molecular changes in most disease models is limited. Here, we conducted transcriptome analysis of murine ocular tissue transduced with recombinant Adeno-associated viruses (AAVs) expressing either human VEGF-A, TNF-α, or IL-6. VEGF expression led to a distinct regulation of extracellular matrix (ECM)-associated genes. In contrast, both TNF-α and IL-6 led to more comparable gene expression changes in interleukin signaling, and the complement cascade, with TNF-α-induced changes being more pronounced. Furthermore, integration of single cell RNA-Sequencing data suggested an increase of endothelial cell-specific marker genes by VEGF, while TNF-α expression increased the expression T-cell markers. Both TNF-α and IL-6 expression led to an increase in macrophage markers. Finally, transcriptomic changes in AAV-VEGF treated mice largely overlapped with gene expression changes observed in the oxygen-induced retinopathy model, especially regarding ECM components and endothelial cell-specific gene expression. Altogether, our study represents a valuable investigation of gene expression changes induced by VEGF, TNF-α, and IL-6 and will aid researchers in selecting appropriate animal models for retinopathies based on their agreement with the human pathophysiology.

## Introduction

Retinopathies such as diabetic retinopathy (DR), age-related macular degeneration (AMD), uveitis or retinopathy of prematurity (ROP) display complex pathologies in patients including vasculopathies, inflammation, neurodegeneration, and fibrosis, ultimately leading to blindness. A number of molecular changes have been associated with these retinal pathologies, for example changes in expression of Vascular Endothelial Growth Factor (VEGF), Tumor Necrosis Factor alpha (TNF-α), or Interleukin-6 (IL-6). VEGF causes vascular leakage and pathological neovascularization, and VEGF protein has been shown to be upregulated in wet AMD^1^, DR^2^, Diabetic Macular Edema (DME)^3^ and ROP^4^. Accordingly, anti-VEGF treatment has emerged as standard-of-care for wet AMD^5^ and DME^6^. Another common hallmark of many retinopathies is inflammation: proinflammatory cytokines such as TNF-α as well as IL-6 proteins are upregulated in the vitreous of DR^7,8^ and uveitis patients^9,10^.

Various pre-clinical rodent models have directly or indirectly shed light on the function of VEGF, TNF-α, or IL-6 in retinopathies. One frequently used animal model displaying vascular pathologies, similar to those observed in proliferative retinopathies such as wet AMD or ROP, is the oxygen induced retinopathy (OIR) model^11,12^. Transgenic mice expressing VEGF^13–15^, intraocular injection of recombinant VEGF protein^16–18^ or recombinant adeno-associated viruses (AAVs) expressing VEGF^19–23^ further demonstrated that VEGF is not only necessary but also sufficient to cause vasculopathies. Accordingly, anti-VEGF treatment prevents neovascularization in the OIR model^17,24^ and these preclinical studies paved the way for modern anti-VEGF therapies. Furthermore, inflammatory processes observed in patients of retinopathies can also be modeled in rodents, for example by uveitis mouse models such as endotoxin- or antigen-induced uveitis^25–27^ or transgenic mice lacking the *Aire* gene^28,29^. Similarly, recombinant proteins or AAV-mediated expression of TNF-α and IL-6 induces inflammation in the rodent eye, although the direct function of IL-6 is more controverse^19,30–32^. Again, anti-TNF-α or anti-IL-6 treatment improves the induced pathologies in diverse uveitis models^33–35^.

In the context of pre-clinical research, AAV mediated gene transfer has recently emerged as a powerful method to create novel animal models. As a main benefit, AAVs allow for long-term expression of a transgene in a tissue- and cell-type-specific manner. Previously, we have shown that AAV-mediated expression leads to constant and long-term expression of human transgenes at 1, 3 and 6 weeks after IVT-injection^19^. We further demonstrated that AAV-mediated expression of human VEGF, TNF-α, and IL-6 in the murine eye leads to pathway-specific, human-relevant retinal pathologies. In brief, AAV-driven expression of VEGF induced vascular leakage and neovascularization. On the other hand, pro-inflammatory cytokines TNF-α and IL-6 both activated immune cells. TNF-α additionally led to vasculitis, fibrosis, and development of fibrotic epiretinal membranes.

In the recent years, next generation sequencing techniques allowed for a detailed investigation of the molecular changes in retinopathies and brought about a better understanding of disease progression in AMD and DR patients^36–38^. Similarly, the transcriptome of multiple rodent models of retinopathies has been sequenced and analyzed^39–42^. For the OIR model, several studies have identified time point-dependent gene expression changes related to hypoxia, angiogenesis, and inflammation^43–47^. However, comparisons with other mouse models of retinopathies are still missing. Additionally, modern single cell RNA-sequencing (scRNA-Seq) approaches gave unprecedented insights into the cellular organization of healthy and diseased mammalian ocular tissues^48–54^. In more detail, Heng et al. demonstrated by single cell sequencing of *Aire*^-/-^ mice, a spontaneous uveoretinitis model, that a very diverse population of immune cells invades the retina of *Aire*^-/-^ mice and that Th1 cells represent the main effector T cells in this model, highlighting the great potential of such single cell sequencing approaches. As the number of available bulk and single-cell datasets in the context of retinopathies increases, so does the potential to integrate data from various retinopathy models and compare them on a molecular level.

In this study, we provide transcriptomic analysis of intravitreally injected recombinant AAVs expressing human VEGF, TNF-α, or IL-6 in mice. We observed that AAV-mediated expression of human transgenes introduced distinct transcriptomic responses: While TNF-α and IL6 displayed overlapping gene expression changes, VEGF overexpression led to a more distinct response compared to TNF-α and IL-6. In more detail, investigating pathways affected by each experimental condition, VEGF led to a specific regulation of extracellular matrix (ECM)-related genes, while TNF-α and IL-6-induced changes in interleukin signaling. We further identified specific gene expression changes associated with TNF-α that included cellular adhesion molecules, such as Madcam1 and we further demonstrated a conserved regulation of MAdCAM-1 by TNF-α in human retinal endothelial cells. By integration of single cell RNA-Sequencing data, we could show an increase of T-cell-specific genes following TNF-α expression and, indeed, TNF-α mediated T-cell invasion was validated by immunofluorescence. In addition to assessing transcriptome changes in the various transgenic mice models, we generated a detailed time-course of transcriptome changes in the established OIR model. Comparing transcriptome changes in AAV overexpression models and OIR mice, we observed the largest overlap between AAV-VEGF treated eyes and the late response (P16) in the OIR model, most prominently including changes in the ECM pathway and endothelial cell specific marker genes. Prominently, OIR-specific changes in gene regulation included neuronal signaling pathways which were not observed after AAV-VEGF transgene expression. In conclusion, our study suggests that each animal model produces distinct gene expression profiles and a careful selection of models according to the requirements of different research questions is necessary.

## Results

### AAV treatment shows distinct gene expression responses to different human transgenes

**Figure 1:**
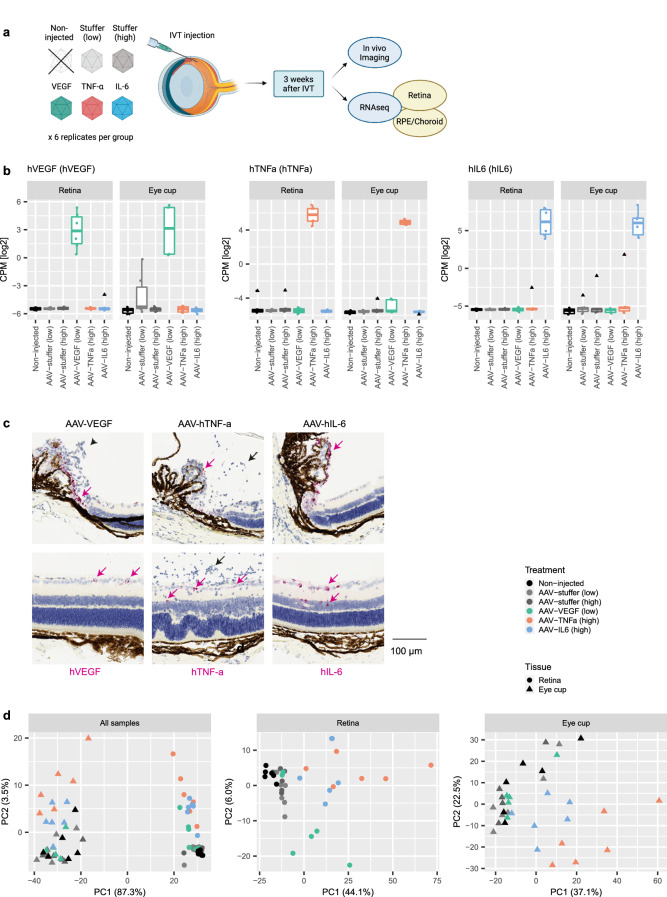
AAV-mediated expression of human VEGF, TNF-α, or IL-6 leads to distinct transcriptome changes. (**a**) Experimental design: Retina and eye cup tissue for RNA sequencing from 6 treatment groups (Non-injected, AAV-stuffer low, AAV-stuffer high, AAV-VEGF, AAV-TNF-α, and AAV-IL-6) were collected 3 weeks after IVT and in vivo imaging. The image was generated using BioRender. (**b**) Specific expression of human transgenes VEGF, TNF-α, and IL-6 in the respective treatment groups in both retina and eye cup tissue (CPM: Counts Per Million). (**c**) In situ hybridization using RNAscope technology demonstrated expression of the three human transgenes (pink arrows) in the ciliary body (upper panel) and the inner layer of the central retina (lower panel). Note the abnormal endothelial cells around the ciliary body in AAV-VEGF treated eyes (black arrowhead) and the infiltrating immune cells in AAV-TNF-α treated eyes (black arrow). (**d**) PCA demonstrated large differences between tissues (upper panel) and specific changes induced by VEGF, TNF-α, and IL-6, respectively, within each tissue (lower panels). Low dose = 1 × 10^8^ VG/eye; High dose = 1 × 10^9^ VG/eye. Percentage of variance explained by the two first principal components is shown together with the respective x- and y-labels.

To investigate and compare transcriptomic profiles of ocular tissues expressing human VEGF, TNF-α, and IL-6, we intravitreally injected mouse eyes with AAVs expressing one of the three human transgenes (Fig. 1a). As controls, we used AAV-stuffer injected eyes at matching concentrations and non-injected eyes. Note that AAV-VEGF was injected at a lower viral dose (1 × 10^8^ VG/eye), while AAV-TNF-α and AAV-IL-6 were injected at 1 × 10^9^ VG/eye. Eyes of all animals were imaged in vivo directly before tissue dissection and RNA sequencing to validate expected pathologies and grade phenotype severity. In line with our previously published study^19^, AAV-stuffer injections did not induce obvious retinal pathologies at both concentrations based on Blue Autofluorescence (BAF) imaging (Supplementary Fig. 1), Fundus Fluorescein Angiography (FFA, Supplementary Fig. 2), and Optical coherence tomography (OCT, Supplementary Fig. 3), apart from few, irregular brighter or darker areas of unknown origin in the BAF images. AAV-VEGF injection in 3 out of 6 samples led to enlarged and abnormally growing vasculature and vascular leakage as seen in the FFA images (Supplementary Fig. 2). AAV-TNF-α injected eyes exhibited cellular infiltrates in the vitreous as shown in the OCT scans (Supplementary Fig. 3). Finally, AAV-IL-6 injected eyes demonstrated subretinal hyperfluorescent foci in BAF imaging (Supplementary Fig. 1).

From all six treatment groups total RNA was extracted from the retina and the posterior eye cup, including retinal pigment epithelium (RPE), choroid, sclera and the ciliary body. Apart from two samples that were excluded due to a low RNA integrity number (RIN), six biological replicates per group were sequenced. Sequencing libraries were sequenced at an average depth of 31.3 million reads, with 80.1% mapping to mRNA transcripts and low ribosomal content (2.1%), suggesting overall good quality of the RNA sequencing data. As expected, human transgenes showed strong expression in their respective treatment groups (Fig. 1b). Although the ShH10 capsid used in this study is described to primarily infect Müller glia^55^, we noticed comparable expression levels of transgenes in both retinal and eye cup, which for the VEGF transgene we confirmed by qRT-PCR (Supplementary Fig. 4a). To identify any major cell types which were transduced by the ShH10 capsid, we performed in situ* hybridization* using RNAscope technology on histological cross-sections of mouse eyes injected with AAV-VEGF, AAV-TNF-α, and AAV-IL-6 (Fig. 1c). In line with the literature^55^, strong and specific expression of transgenes was limited to the inner part of the retina (presumably retinal ganglion cells and Müller glia). Since no mRNA staining was observed in AAV-stuffer control treated eyes (Supplementary Fig. 4b), we concluded that probes used to detect the three respective transgenes were specific. Within the eye cup, no expression of human transgenes was observed in the RPE or choroid tissue, however, we noticed strong expression in the ciliary body, indicating that the gene expression measured by RNA-Seq in the eye cup samples originated from the ciliary body (Fig. 1c).

Principal component analysis (PCA) indicated the largest variance in gene expression between retina and eye cup samples (Fig. 1d, left panel). Within the retinal tissue we observed further separation of samples between the main three treatment groups and both AAV-stuffer controls or non-injected controls (Fig. 1d, middle and right panel). In the PCA, samples from all three control groups appeared in close vicinity, suggesting no major changes in gene expression caused by IVT injection of AAVs per se. Further, AAV-TNF-α and AAV-IL-6 samples grouped together and displayed good separation from control samples in both retina and eye cup tissue. AAV-TNF-α samples however, showed an overall stronger separation from controls compared to AAV-IL-6. In case of AAV-VEGF, we found four of the AAV-VEGF retina samples to be clearly distinct from control and AAV-TNF-α/AAV-IL-6 samples (Fig. 1d). The remaining two retinal samples (replicate 2 + 3) of AAV-VEGF treated mice, however, showed no separation from controls within the first two principal components (Supplementary Fig. 4c,d). In line with these findings, both replicates 2 + 3 appeared normal based on the FFA (Supplementary Fig. 2; Supplementary Fig. 4c), suggesting that virus transduction may have been insufficient to cause a phenotype. As we further observed reduced expression of human VEGF in both samples (Supplementary Fig. 4c), we removed these two samples from all subsequent analysis.

### Differential gene expression analysis reveals overlapping responses upon AAV-TNF-α and AAV-IL-6 treatment

**Figure 2:**
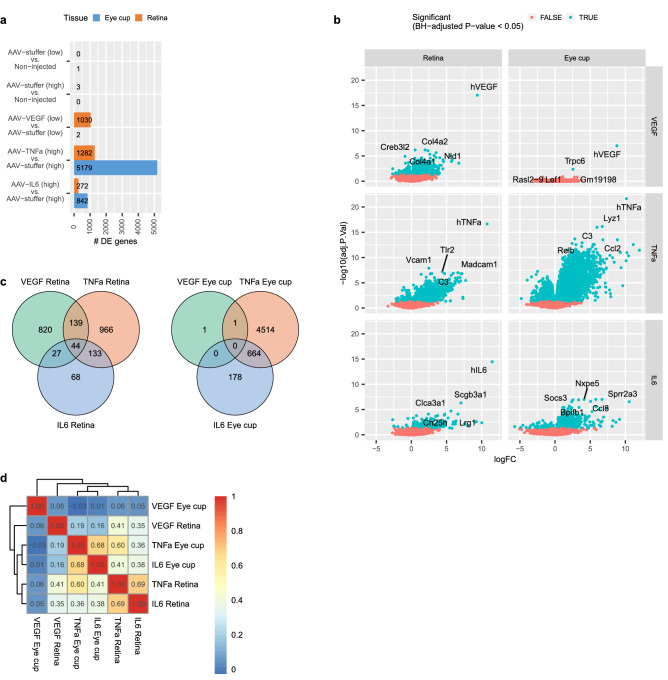
Differential gene expression analysis revealed related changes in TNF-α and IL-6 treated eyes compared to VEGF. (**a**) Numbers of differentially expressed genes in each treatment compared to their respective controls (BH-adjusted p-value < 0.05). (**b**) Volcano plots indicating top differentially regulated genes in retina and eye cup tissue upon injection with AAV-VEGF, AAV-TNF-α, or AAV-IL-6. (**c**) Venn diagrams representing sets of differentially expressed genes overlapping between treatment groups. 820 genes were specifically regulated by AAV-VEGF. Large overlap between TNF-α and IL-6-induced differentially regulated genes in both retina and eye cup tissue. (**d**) Spearman correlation verified a strong correlation between TNF-α and IL-6-induced gene expression changes in both retina and eye cup tissue. Dendogram shows hierarchical clustering and therefore similarity of treatment induced fold-changes compared to their respective controls.

Next, we investigated differentially expressed (DE) genes in the three treatment groups AAV-VEGF, TNF-α, and AAV-IL-6 in comparison to their respective AAV-stuffer control, as well as AAV-stuffer compared to non-injected controls (Supplementary Table S1). As expected, we found only few significant gene expression changes between AAV-stuffer injected and non-injected samples for both retina and eye cup (BH-adjusted p-value < 0.05; Fig. 2a), once again suggesting limited impact of control AAV injection on gene expression. We observed the largest number of significant expression changes in TNF-α and IL-6 transfected eye cup tissue, while AAV-VEGF mostly affected retinal gene expression but not the eye cup tissue. In each treatment group, the respective human transgene was among the top significantly differentially up-regulated genes within their corresponding treatment group, validating strong AAV-mediated expression in both retina and eye cup (Fig. 2b). In AAV-TNF-α injected eyes, cellular adhesion molecules such as *Vcam1* and *Madcam1* and chemokines such as *Ccl2* were among the strongest deregulated genes, supporting the well-known function of TNF-α in regulating immune cell adhesion to endothelial cells^56^. To confirm the RNA sequencing results, *Ccl2* expression was validated by qRT-PCR, which demonstrated similar expression patterns with the highest expression of *Ccl2* found in AAV-TNF-α treated eyes (Supplementary Fig. 4e,f). Notably, endogenous VEGF was down-regulated in AAV-VEGF treated eyes and endogenous TNF-α upregulated upon AAV-mediated expression of TNF-α or IL-6 (Supplementary Fig. 5).

To find common regulators of retinal disease, but also identify specific pathways that can be correlated to the specific phenotypes induced by the three expressed transgenes, we compared transcriptomic signatures of AAV-VEGF, AAV-TNF-α, and AAV-IL-6 (Fig. 2c). Overall, in retina tissue only 44 genes were significantly differentially regulated in all three AAV-treatment groups. 820 DE genes were specific to AAV-VEGF treated retina, further highlighting a different transcriptional response in AAV-VEGF compared to AAV- TNF-α and AAV-IL-6. Only 2 genes were significantly differentially regulated in AAV-VEGF treated eye cup tissue, suggesting that VEGF mainly affects retinal cells in the presented AAV-driven model. 1282 and 272 genes were DE in AAV-TNF-α and AAV-IL-6 transduced retinae, respectively, with 133 genes overlapping between these two groups. Furthermore, only 68 genes were specifically regulated by AAV-IL-6, but 966 by TNF-α in the retina, suggesting that a large part of gene expression changes induced by AAV-IL-6 were included in the AAV-TNF-α response. In line with these results, the highest correlation of fold changes was observed between AAV-TNF-α and AAV-IL-6 treated expression profiles in both retina and eye cup tissue, while AAV-VEGF appeared more distinct (Fig. 2d).

### Pathway enrichment analysis highlights changes in the extracellular matrix for AAV-VEGF and a strong immune reaction in AAV-TNF-α and AAV-IL-6 treated eyes

**Figure 3:**
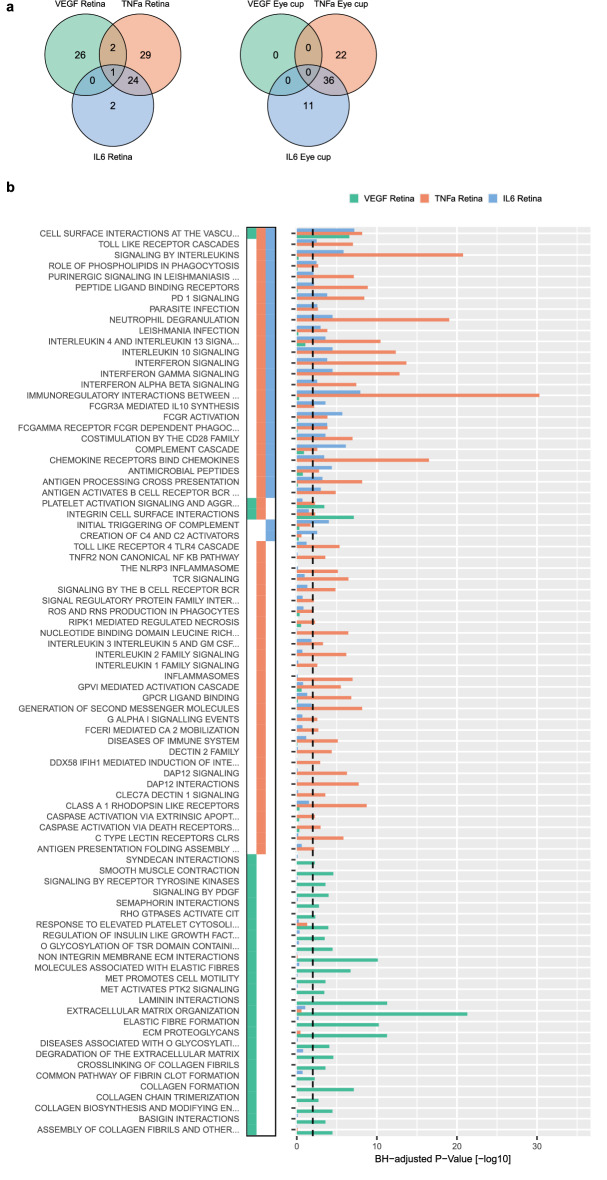
AAV-VEGF treatment altered ECM-related genes, while AAV-TNF-α and AAV-IL-6 induced a strong inflammatory response in retinal tissue. (**a**) 33 pathways were specifically regulated by VEGF in the retina and a majority of pathways regulated by IL-6 was also regulated by TNF-α in both retina and eye cup tissue. (**b**) All REACTOME pathways significantly enriched (BH-adjusted p-value < 0.01) in at least one of the treatments (AAV-VEGF, AAV-TNF-α, and AAV-IL-6).

Next, we analyzed REACTOME pathways significantly affected (hypergeometric test, BH-adjusted p-value < 0.01) by AAV-VEGF, AAV-TNF-α or AAV-IL-6 treatment. Only 1 pathway was enriched in the retina of all three treatment groups (Fig. 3a), namely “Cell Surface Interactions at the Vascular Wall” (Fig. 3b). In AAV-VEGF injected retinae, the top de-regulated pathway was “Extracellular Matrix Organization”, while AAV-TNF-α and AAV-IL-6 both induced the strongest gene expression changes in the pathway “Immunoregulatory Interactions Between a Lymphoid and a Non-Lymphoid Cell”. Among VEGF specific pathways in the retina, “ECM Proteoglycans” and Collagen-related pathways were significantly deregulated. A total of 29 pathways were specifically regulated in TNF-α injected retinal tissue, for example the “Class A 1 Rhodopsin Like Receptors” pathway. Two pathways were enriched specifically in AAV-IL-6 samples: “Initial Triggering of Complement” and “Creation of C4 and C2 activators”. Pathways regulated by both TNF-α and IL6 included “Signaling by Interleukins”, and the “Complement Cascade” pathway.

Since only very few genes were significantly differentially regulated in AAV-VEGF transduced eye cup tissue, only AAV-TNF-α and AAV-IL-6 treatment was compared in the eye cup tissue (Supplementary Fig. 6). Once again, similar to the differential gene expression analysis, the majority of pathways identified in AAV-IL-6 eyes were shared with AAV-TNF-α in both retina and eye cup tissue (Supplementary Fig. 6b).

### AAV-TNF-α mice show strong activation of Complement pathway which can be blocked by anti-TNF- α treatment

**Figure 4:**
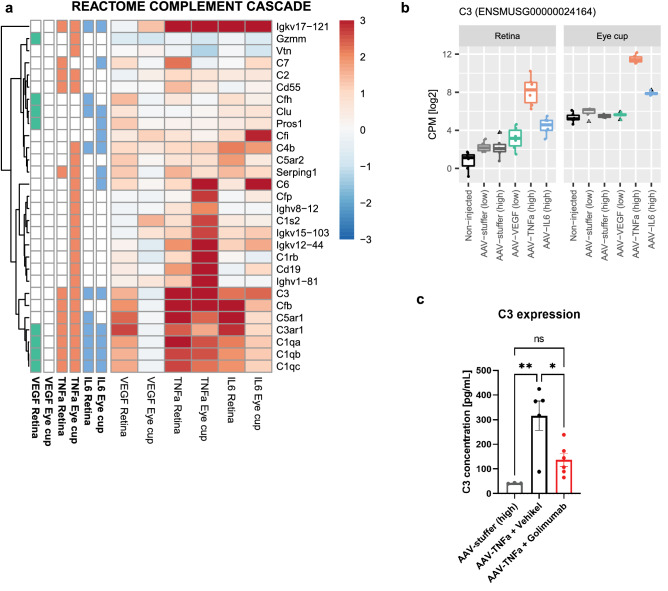
TNF-α induced a strong upregulation of the complement pathway (**a**) Upregulation of several members of the complement cascade pathway was observed especially in AAV-TNF-α transduced eye cup tissue. Colored squares in columns on the left indicate significant gene expression changes with an BH adjusted P-value < 0.05. (**b**) C3 expression was highly upregulated in AAV-TNF-α treated retina and eye cup. (**c**) C3 protein was also upregulated by AAV-TNF-α in an independent mouse cohort at 6 weeks after AAV-TNF-α treatment and this upregulation was partially rescued by anti-TNF-α treatment with golimumab (**p < 0.01; *p < 0.05, 1-way ANOVA with Tukey post-hoc test; n = 3–6) as measured by ELISA.

The complement cascade pathway is potentially involved in the disease progression for diverse retinopathies including AMD^5^. We therefore analyzed gene expression changes in this pathway in more detail. Comparing all three treatment groups in both tissues, the highest absolute number of DE genes associated with the REACTOME pathway “Complement Cascade” was observed in the eye cup tissue of AAV-TNF-α treated eyes (Fig. 4a). Interestingly, the complement activators C3, Cfp and Cfb were strongly upregulated in AAV-TNF-α, while complement inhibitors such as Cfh or Cfi were not regulated in AAV-TNF-α. In contrast, fewer complement-related genes were regulated by AAV-IL-6 and included a strong upregulation of the complement inhibitor Cfi, indicating no or milder activation of the complement pathway compared to AAV-TNF-α. Overall these results suggest an activation of the complement pathway in AAV-TNF-α treated eyes. One of the genes showing largest upregulation in AAV-TNF-α injected eyes was C3 in both retina and eye cup tissue (Fig. 4b). To validate our results, we measured C3 expression by ELISA in an independent mouse cohort^57^ and verified the upregulation of also C3 protein at 6 weeks after AAV-TNF-α treatment (Fig. 4c). Furthermore, as a proof-of-mechanism, the TNF-α neutralizing antibody golimumab significantly reduced TNF-α-mediated upregulation of C3 (Fig. 4c), demonstrating that C3 upregulation is dependent on TNF-α and may be reversed in our model.

### Cell adhesion molecules including Madcam-1 are specifically regulated by TNF-α

**Figure 5:**
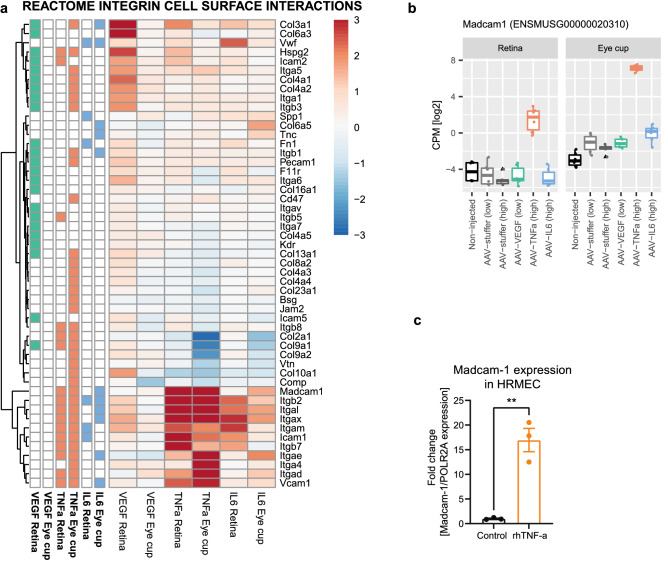
Cell adhesion molecules including Madcam-1 are specifically upregulated in AAV-TNF-α treated eyes. (**a**) Specific gene expression pattern within the REACTOME pathway “Integrin Cell Surface Interactions” revealed an upregulation of Collagen genes by AAV-VEGF. Left columns again indicate significant gene expression change with BH-adjusted P-value < 0.05. Members of the integrin family were mostly affected by AAV-TNF-α and AAV-IL-6 treatment, but not AAV-VEGF. (**b**) Madcam1 was one of the most upregulated genes in AAV-TNF-α injected eyes in both retina and eye cup tissue. (**c**) Human MADCAM1 expression is upregulated by TNF-α stimulation of human retinal microvascular endothelial cells (HRMECs, **p < 0.01, unpaired t-test, n = 3).

Integrin Cell Surface Interactions was among the few pathways significantly changing in AAV-VEGF and AAV-TNF-α treatment groups in the retina, thus, we were interested which gene expression signatures are common or different between these two conditions. Within the Integrin Cell Surface Interaction pathway, we observed 4 different clusters: genes that were similarly upregulated in both AAV-VEGF and AAV-TNF-α, AAV-VEGF-specific upregulated, AAV-TNF-α specific down-regulated, and regulated in both AAV-TNF-α and AAV-IL-6 (Fig. 5a). In the retina, several collagens were upregulated specifically in AAV-VEGF treated eyes, while AAV-TNF-α and AAV-IL-6 both induced expression of genes encoding for integrin subunits. Interestingly, cellular adhesion molecules (CAMs), such as *Icam1*, *Vcam1* and *Madcam1* were highly upregulated only in AAV-TNF-α injected eyes (Fig. 5a,b). Madcam1 has been linked previously to TNF-α and is an important player in the development of inflammatory bowel diseases, but only few studies describe Madcam1 in the context of retinopathies, in contrast to other adhesion molecules like Vcam1. Thus, to test whether MAdCAM-1 may also be relevant for human retinopathies, we stimulated primary human retinal microvascular endothelial cells (HRMEC) with recombinant, human TNF-α. Remarkably, TNF-α stimulation strongly increased *Madcam1* expression in HRMECs (Fig. 5c), suggesting that MAdCAM-1 may play an important role also in the human retina and may contribute to disease progression of various human retinopathies.

### Monocyte-, B- and T-cell specific genes are enriched in AAV-TNF-α treated eyes

**Figure 6:**
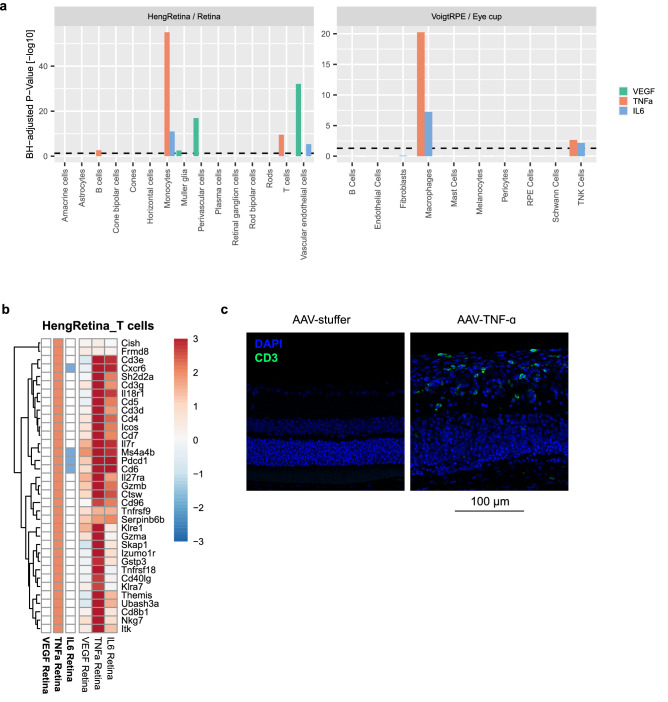
Endothelial-cell specific marker genes are upregulated by VEGF and T-cell-specific genes by TNF-α. (**a**) VEGF expression induced upregulation of endothelial and perivascular cell specific marker genes in retinal tissue. Both TNF-α and IL-6 expression induced an enrichment of macrophage/monocyte-specific genes. Only TNF-α induced significant upregulation of B- and T-cell-specific genes in the retina. (**b**) Heatmap of T-cell-specific genes significantly deregulated in any treatment demonstrated a strong upregulation of these genes by AAV-TNF-α. (**c**) Immunofluorescence staining with the pan-T-cell marker CD3 revealed T-cell infiltration in AAV-TNF-α injected eyes (green = CD3, blue = DAPI). Note the increased retinal thickness and disorganization of retinal layers in AAV-TNF-α treated eyes.

As bulk RNA-Seq data does not resolve gene expression on a cell-specific level, we made use of recently published human retinal and RPE single cell RNA-Seq studies^48,49,54^ in order to identify cell type-specific changes induced by AAV-VEGF, AAV-TNF-α, and AAV-IL-6. For this purpose, we identified cell-specific marker genes for different cell types in the scRNA-Seq datasets of both retina and RPE tissue and compared these to the differentially expressed genes in our AAV-induced retinopathy model. In case of the Heng single cell RNA-Seq data, we combined samples of wildtype mice and uveitis-like *Aire*^*-/-*^ retinae to best reflect all cell types present in the healthy, but also inflamed retina, akin to our AAV-TNF-α and AAV-IL-6 treated eyes. In line with the vasculopathies observed in AAV-VEGF-treated eyes, a significant enrichment (Hypergeometric test, BH-adjusted p-value < 0.05) of endothelial and perivascular cell-specific marker genes after AAV-VEGF treatment was observed (Fig. 6a, left panel). In contrast, monocytes/macrophages-specific genes significantly overlapped with DE genes from both AAV-TNF-α and AAV-IL-6 treated retina and eye cup tissue (Fig. 6a). Interestingly, enrichment of these immune cells was stronger for AAV-TNF-α injected eyes compared to AAV-IL-6 injected eyes, matching with more severe inflammation induced by AAV-TNF-α described previously^19^. Follow up analysis with a second available scRNA-Seq dataset^49^ from human retina and RPE tissue validated significant enrichment of macrophage/microglia specific markers in AAV-TNF-α, and AAV-IL-6 derived DE genes, as well as the overlap between endothelial cell type-specific markers and DE genes identified in AAV-VEGF treated eyes (Supplementary Fig. 7a).

Expression changes of the identified relevant cell type-specific marker genes (vascular endothelial cells, monocytes) were mostly positive (Supplementary Fig. 7b,c), suggesting a general increase of these cell types upon the respective AAV treatment. Our enrichment analysis further indicated the strongest upregulation of T-cell specific marker genes in TNF-α retinae (Fig. 6a,b). We followed up on this observation by histological analysis of retinal cross-sections, and indeed validated that cells expressing the pan-T-cell marker CD3 were present in AAV-TNF-α injected eyes (Fig. 6c), suggesting that T-cell infiltration is induced by AAV-TNF-α.

### Gene expression changes induced by VEGF overexpression are similar to those observed in the oxygen induced retinopathy model

**Figure 7:**
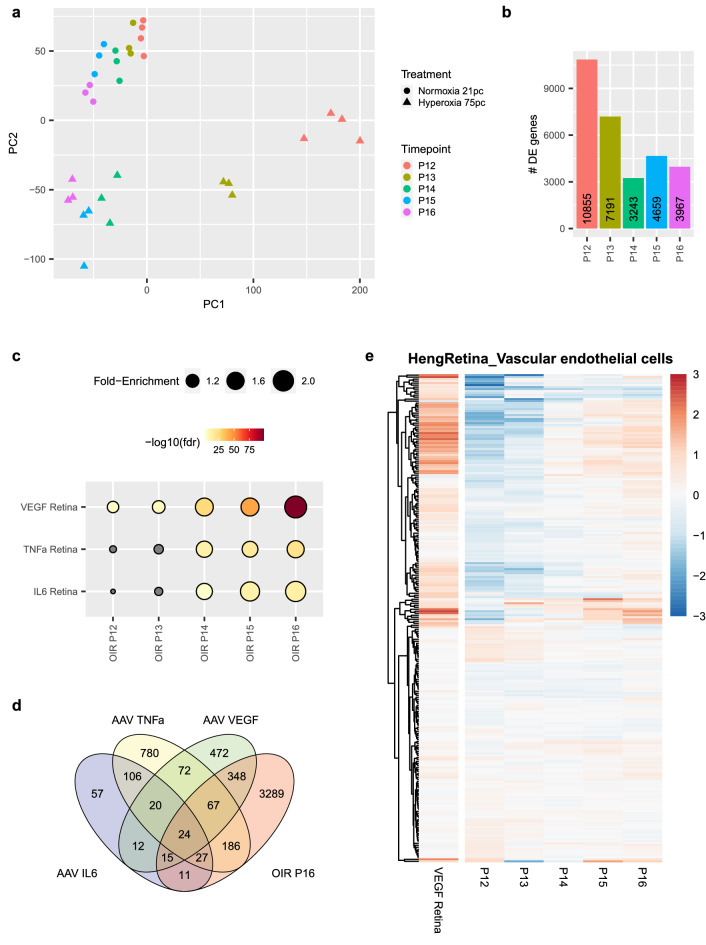
Transcriptomic signature of AAV-VEGF treatment is most similar to that observed in P16 OIR mice. (**a**) PCA demonstrated a clear separation of samples according to treatment (normoxia vs. hypoxia) and timepoint. (**b**) The largest number of gene expression changes was observed at P12 and P13 and the number of differentially regulated genes stabilized at 3000–5000 genes at P14-P16. (**c**) The strongest enrichment for similar gene expression pattern was observed between OIR P16 and AAV-VEGF (2.2 fold-enrichment). (**d**) Overlap between DE genes of AAV-VEGF, AAV-TNF-α, AAV-IL6 and the OIR model (P16) are depicted in the Venn diagram. (**e**) Genes specifically expressed by endothelial cells were upregulated in AAV-VEGF injected eyes and P15/P16 OIR retina, but not P12/P13 OIR retina.

A frequently used animal model for proliferative retinopathies is the oxygen induced retinopathy (OIR) model, presenting both neovascularization and neurodegeneration. Neonatal mice are exposed to 75% oxygen from postnatal day 7 to 12, and thereafter return to room air (21% oxygen) until day 17. At day 7, the retinal vasculature of mouse pups is still immature and vulnerable to high oxygen conditions. Loss of capillaries in the center of the retina leads to the formation of an avascular area. Upon return to room air at day 12 the area becomes hypoxic and Hif1α dependent VEGF expression has been shown to trigger angiogenesis, peaking at P17^11,12^. Due to the hypoxia, also apoptosis of neurons and retinal thinning is observed in the avascular area in this model^58^. To better understand the transcriptome changes induced by AAV-VEGF, TNF-α, and IL-6, we aimed to compare the transcriptome of AAV-transduced retinae to OIR retinae. First, we looked at the transcriptome changes induced in the OIR model compared to controls at postnatal day 12 (P12), P13, P14, P15 and P16. PCA demonstrated a clear separation between the OIR model and controls (Fig. 7a). Remarkably, also time-dependent changes in the transcriptome of control and OIR mice were clearly visible in the PCA (Fig. 7a). The largest number of significantly differentially expressed genes (BH-adjusted p-value < 0.05) were detected at P12 (10,855), shortly after mice were removed from the hyperoxic chamber (Supplementary Table S2). At later timepoints the number of differentially expressed genes decreased to around 3000–7000 genes between P13 and P16 (Fig. 7b), suggesting a transient response directly after the end of the hyperoxic treatment (P12). As expected, starting at P13 at the onset of hypoxia in the avascular area, endogenous expression of the Hif1a-dependent factor mouse *Vegf* gradually increased with time (Supplementary Fig. 8a), similar to previous studies^43,59^. *Il6* expression was not detected, while *TNF-α* expression peaked at P13 and 14 (Supplementary Fig. 8b), suggesting an early inflammatory component in the OIR model.

When comparing the three AAV and OIR mouse models, we observed a significant overlap of differentially expressed genes (Hypergeometric test, BH-adjusted p-value < 0.01) in OIR retina compared to those identified in the AAV-VEGF model (Fig. 7c,d). The largest enrichment of DE genes was found between AAV-VEGF mice and the OIR model at P16, which is the peak time of the vascular phenotype in the OIR model. Integration of the previously described single cell data sets^48,54^ demonstrated that, among others, also vascular cell-specific marker genes were enriched in the OIR data set (Supplementary Fig. 8c) similar to the AAV-VEGF data. Remarkably, the majority of endothelial cell-specific genes was initially down-regulated in the early OIR time points, and then upregulated starting from P15, similar to AAV-VEGF (Fig. 7e). In contrast, DE genes identified from the late-stage OIR mouse model, but not those identified from AAV-VEGF, significantly overlapped with genes specifically expressed in bipolar cells. We concluded that the perivascular and vascular endothelial cell types are affected by AAV-VEGF treatment (Fig. 6a) and additional cell types are affected in the OIR model due to malnutrition and ischemia induced in this model.

Finally, we aimed to understand which pathways were affected in the AAV-VEGF driven retinopathy models compared to the OIR model (Supplementary Fig. 8d). Interestingly, multiple pathways relating to the extracellular matrix were enriched in DE genes identified from either the AAV-VEGF or the OIR model, suggesting similar changes in both models. Once again, “Extracellular Matrix Organization” appeared as one of the strongest de-regulated pathways in both AAV-VEGF but also OIR P16. Interestingly, neuronal signaling-related genes were enriched among the OIR-specific pathways, while AAV-VEGF-specific pathways included pathways involved in cell surface interactions. Altogether, our analysis revealed similarities but also differences between the four different mouse models for retinopathies and, thus, is a valuable resource for researchers to identify the most appropriate animal model with respect to their pre-clinical research topic.

## Discussion

In this study, we measured transcriptome changes induced by AAV-VEGF, AAV-TNF-α, and AAV-IL-6 and compared these expression changes with each other and with gene expression changes observed in the well-established OIR model. Our aim was to further understand the potential molecular mechanisms contributing to retinal pathologies, but also to increase our ability to select the appropriate animal models for each pathology.

AAVs have emerged as a useful tool to quickly generate novel animal models that allow a high degree of flexibility regarding timing and tissue-specific expression. In our study, we chose the ShH10 capsid for expression of the three human transgenes. Even on a global scale, measured via a high-throughput method such as RNA-Seq, injection of the control capsid (1 × 10^8^ or 1 × 10^9^ VG/eye) into mouse eyes with a biologically inactive sequence, produced very few gene expression changes. AAV-mediated expression of hTNF-α, hIL-6, or hVEGF, led to high expression of these human transgenes, which in turn produced strong and distinct transcriptome changes. Although measurement errors in RNA-Seq caused by the simultaneous presence of human and murine versions of the AAV-expressed transgene are theoretically possible, we expect no large mis-quantification, since human transgenes are codon optimized and possess a V5 tag, hence they are sufficiently dissimilar to their endogenous transcript sequence. In addition, we did not observe any reduction in the percentage of uniquely mapped reads in the presence of AAV expressed transgenes (data not shown).

According to the present literature, ShH10 primarily infects Müller glia^55^, which anatomically span the complete retina with their processes, thereby allowing for secretion of VEGF, TNF-α, and IL-6 throughout the retina. RNAscope analysis indicated expression of human transgenes within the RGC layer and inner nuclear layer, potentially deriving from Müller glia. Moreover, RNAscope analysis further demonstrated strong expression of all three transgenes in the ciliary body, explaining why high expression levels of the transgenes were detected in RNA-Seq analysis of the dissected eye cup tissue including the ciliary body. Strong expression of human transgenes in the ciliary body might also explain the growth of endothelial tissue around the ciliary body in the peripheral retinal areas in AAV-VEGF injected eyes^19^. Interestingly, in the original paper describing the ShH10 capsid, IVT injection of ShH10-GFP led to strong GFP expression in Müller glia as well as the ciliary body, but this finding was not further discussed^55^. Meanwhile, other researchers have investigated AAV capsids with good transduction efficiency of the ciliary body for treatment of glaucoma and found the ShH10 capsid most suitable to transduce the ciliary body compared to AAV1, AAV2, AA5 and AAV6 capsids^60^. While transduction of the ciliary body may be desirable for certain applications, strictly cell type-specific expression may be needed e.g. for gene therapy approaches delivering intracellular proteins. For secreted factors, such as VEGF, TNF-α, and IL-6, cell type-specific expression may not be required. However, it will still be interesting in the future to test other capsids to express VEGF, TNF-α, and IL-6 in the retina and compare the phenotypes achieved by different cell tropisms. Thus, we conclude that careful selection of capsid should be done for each application.

In our analysis, significantly changing genes present in the complement cascade were strongly and specifically upregulated in eyes injected with AAV-TNF-α. Among them, the complement pathway factor C3 was one of the strongest upregulated genes in AAV-TNF-α injected eyes. C3 plays a central role in the activation of the classical and alternative complement pathway and mutations in C3 and other members of the complement cascade correlate with higher risk of AMD^61–63^ and uveitis^64^. Further it is known that C3 is upregulated in the retina of AMD patients^65,66^, as well as vitreous, aqueous and serum of uveitis patients^67–69^, thus, targeting the complement system presents an attractive approach to treat retinopathies^70^. Accordingly, anti-C3 and anti-C5 treatment are currently evaluated in late-stage clinical trials for geographic atrophy (GA) secondary to AMD^71,72^. In the past, transgenic animals modifying the expression of complement genes, or mice expressing human variants of complement-related genes, have proven extremely useful to understand their contribution to disease progression^65,73–78^. To further develop novel and effective therapies, animal models with robust activation of the complement pathway are needed. In LPS-induced uveitis model, complement-related genes including C3 are upregulated^39^, suggesting complement activation. Similarly, experimental autoimmune uveitis (EAU) leads to activation of the complement system and blockage of the complement system ameliorates disease pathology^79^. Activation of the complement system is also visible in the laser-induced choroidal neovascularization (CNV) model, and inhibition of various complement factors had beneficial effects^80–82^. However, both the experimental uveitis models and laser CNV model are transient and only allow for short-term investigations. In contrast, in the presented AAV-TNF-α model, C3 showed strong and sustained upregulation at 3 and 6 weeks after IVT injection of AAV-TNF-α, thus, allowing long-term studies of the complement system. In addition, as a proof-of-mechanism we showed that treatment with the neutralizing TNF-α antibody golimumab reduces C3 expression, demonstrating that complement activation is reversible in our model. In summary, the AAV-TNF-α induced mouse model provides a valuable tool to study the role of the complement system in disease progression, featuring long-term TNF-α and C3 expression, which is comparable to the human pathology that develop over decades.

In addition to C3, cellular adhesion molecules (CAMs), such as *Icam1*, *Vcam1* and *Madcam1* were highly upregulated in AAV-TNF-α injected eyes, strengthening the previous observation of immune cell infiltration and vasculitis in AAV-TNF-α injected eyes by histology^19^. MAdCAM-1 is induced by TNF-α in diverse endothelial cells and recruits T-cells to inflamed tissues^83–85^ and, therefore, has recently been identified as an interesting target to treat inflammatory bowel diseases^86^. In addition to Madcam1 upregulation, we observed T-cell infiltration in our AAV-TNF-α driven uveitis-like model, in line with well-described role for T-cells in uveitis patients and uveitis rodent models^87,88^. Although there is general interest in the role of TNF-α and CAMs in the context of retinopathies, to our knowledge there exists only one recent study by Peng et al*.* demonstrating a direct function for murine Madcam1 in retinal degeneration^83^. In line with Peng et al., we demonstrated upregulation of Madcam1 in AAV-TNF-α injected eyes correlating with an invasion of T-cells. Expanding on this observation, we demonstrated that MAdCAM-1 is also upregulated in TNF-α stimulated HRMECs, suggesting that the TNF-α induced regulation of MAdCAM-1 may also be relevant in human retinal cells.

In order to better understand the differences between various retinopathy disease models, we compared gene expression profiles of the generated AAV-driven retinopathy models to RNASeq data of the frequently used OIR model^11,12^. To capture transient expression dynamics present in the OIR model, we collected mouse eyes at 5 subsequent time points after hyperoxic treatment. Indeed, other authors have performed analysis of OIR gene expression, however, many studies relied on microarray data only assessing part of the transcriptome in contrast to the whole transcriptome analysis presented here. On the other hand, OIR RNA-Seq studies included less dense sampling of timepoints compared to our study, in which we provide more detailed insights into time-dependent gene expression changes. The study of Yang et al.^46^ focused on gene expression changes during and shortly after hyperoxic treatment, while our study was mostly interested in gene expression changes after the return to normoxic conditions and a detailed comparison to AAV induced gene expression changes. Nevertheless, we observed shared gene expression signatures of key genes between the two studies, for example VEGF and Edn2 gene were both upregulated at P13.

It is well known that VEGF is a major driver of the OIR model and anti-VEGF treatment prevents neovascularization in the OIR model^17,24^. Thus, as expected, DE genes in the AAV-VEGF model showed the largest overlap with those identified in the OIR model. By integration of single-cell RNA-Seq expression data, we showed that within the time-course measured for the OIR model, endothelial cell-specific genes were first down-regulated and then up-regulated starting from P15. Also other studies have found downregulation of angiogenesis-related genes such as *CD34* in the OIR model at P12, while endothelial cell-specific genes such as *Esm1* or *Edn2* were upregulated at P17^89^. *Esm1* is a well-known gene induced by VEGF in tip cells with a crucial role in angiogenesis and its blockage inhibits neovascularization of diverse rodent models of neovascularization including the OIR, laser CNV and the rho/VEGF transgenic mouse^90,91^. Accordingly, we also observed upregulation of the *Esm1* gene in both the OIR dataset but also the AAV-VEGF driven model, resembling very well the observed neovascularization phenotype. Since we provide a high time-resolution of gene expression changes in the OIR model, we were able to pinpoint the switch between down- and up-regulation of endothelial cell-specific genes to P14. These gene expression changes fit very well with the phenotypic changes present in the OIR model, where at P12 the development of the vasculature is impaired and avascular areas are present, and later on, neovascularization peaks at around P17^92,93^.

Although many pathways including ECM-related pathways and endothelial cell-specific responses were similar between the OIR model and AAV-VEGF treated mice, we also observed differences between these disease models: For example, in the OIR model, but not AAV-VEGF, neuronal signaling pathways and synapse-related genes were significantly enriched for DE genes. In line with this observation, we observed a significant overlap of neuronal cell-specific genes (e.g. bipolar cells, RGCs) and DE genes identified in the OIR model. For the OIR model pups at age P12 to P17 are used, a timepoint which corresponds to a time-point where vascular and neuronal networks are still developing in the murine retina^94,95^. Furthermore, neuronal death and decreased retinal function has been observed in the OIR model^96,97^, probably due to ischemia and malnutrition induced by hypoxia. Accordingly, the OIR model has been discussed as the more appropriate model for retinopathy of prematurity (ROP)^93^, a disease affecting human preterm infants. While very young mouse pups are needed for the OIR model, old or diabetic mice may be combined with AAV overexpression of human transgenes, to mirror more closely age-related or diabetes-associated human retinopathies such as AMD or DR in the future.

Despite our efforts to ensure optimal experimental design, certain limitations apply, most prominently that AAV and OIR sequencing data sets were generated in independent experiments. Furthermore, also from a biological perspective, the models differ, since the OIR model uses very young mouse pups during development, in contrast to our AAV-induced models that were developed using adult mice. While in the OIR study 3 or 4 samples where included at each time-point, the AAV study was performed with 5 or 6 replicates per group. In addition, library preparation and sequencing was dependent on different protocols. To alleviate these limitations, our analysis is based on relative fold changes between treatment and respective controls, rather than on absolute expression values, thereby accounting for differences in library preparation.

Altogether, here we characterized the gene expression profiles of AAV-VEGF, TNF-α, or IL-6 driven retinopathy mouse models, and combined these measurements with publicly available singe-cell RNA expression data. Our study gives unique insights into the molecular and cell-specific changes leading to retinal pathologies, hereby uncovering novel potential treatment options for retinal diseases, and at the same time offering the means to test them in a stable disease model comparable to the human pathology. By further measuring gene expression changes in the well-established OIR model, we compared new and established animal models for retinopathies and provide researchers with important information guiding the decision of which animal model best suits the need of a given pre-clinical research project.

## Methods

### In vivo* experiments*

C57BL/6 J mice were purchased from Charles River (Sulzfeld, Germany). 6–8-week-old male mice were used for the AAV study. Pregnant females were purchased for the OIR study and male and female pups at P12 were used for the OIR experiments. Mice were housed in individually ventilated cages in groups of 2–5, constant temperature and a 12-h light/dark cycle. Mice had ad libitum access to standard rodent chow and water. Animal experiments were performed in accordance with the German animal welfare act, the guidelines of the Federation of the European Laboratory Animal Science Association (FELASA), the ARRIVE guidelines and were reviewed and approved by the governmental body responsible for animal welfare in the state of Baden Württemberg (Regierungspräsidium Tübingen, Germany).

### AAV production

Plasmids with the ubiquitous CAG promoter were generated encoding for three human, codon optimized transgenes (VEGF-A 165, TNF-α and IL-6) that have a V5-tag sequence at the 3’ end of the gene were generated previously^19^.The AAV-stuffer control construct includes a non-coding sequence from the 3’ UTR of the *UBE3A* gene and has been described elsewhere (Strobel 2015). AAVs used in this study were packaged into the ShH10 capsid (Klimczak 2009). AAV production and quantification by quantitative PCR was done according to previously published protocols (Strobel 2019). The following human codon-optimized sequences were included in each of the recombinant AAVs:

AAV-hIL6—ATGAACAGCTTCAGCACCAGCGCCTTCGGACCTGTGGCTTTTTCTCTGGGACTGCTGCTGGTCCTGCCTGCCGCTTTTCCAGCTCCTGTTCCTCCTGGCGAGGACAGCAAAGATGTGGCCGCTCCTCATAGACAGCCTCTGACCAGCTCCGAGCGGATCGATAAGCAGATCCGGTACATCCTGGATGGCATCAGCGCCCTGCGGAAAGAGACATGCAACAAGAGCAACATGTGCGAGAGCAGCAAAGAGGCCCTGGCCGAGAACAACCTGAACCTGCCTAAGATGGCCGAAAAGGACGGCTGCTTCCAGAGCGGCTTCAACGAGGAAACCTGCCTGGTCAAGATCATCACCGGCCTGCTGGAATTCGAGGTGTACCTGGAATACCTGCAGAACAGATTCGAGTCCAGCGAAGAACAGGCCAGAGCCGTGCAGATGAGCACCAAGGTGCTGATCCAGTTCCTGCAGAAGAAGGCCAAGAACCTGGACGCCATCACCACACCTGATCCTACCACAAATGCCAGCCTGCTGACAAAGCTGCAGGCCCAGAATCAGTGGCTGCAGGACATGACAACCCACCTGATTCTGCGGAGCTTCAAAGAGTTTCTGCAGAGCAGCCTGCGGGCCCTGAGACAAATGGGAGGCGGAGGATCTGGCGGAGGCGGATCTGGAAAGCCCATTCCTAATCCTCTGCTGGGCCTCGACAGCACCTGATGATAA.

AAV-hTNFa—ATGAGCACCGAGAGCATGATCAGAGATGTGGAACTGGCCGAGGAAGCCCTGCCTAAGAAAACAGGCGGACCTCAGGGCAGCAGAAGATGCCTGTTTCTGAGCCTGTTCAGCTTCCTGATCGTGGCAGGCGCCACCACACTGTTCTGTCTGCTGCACTTTGGAGTGATCGGCCCTCAGAGAGAGGAATTCCCCAGAGATCTGTCCCTGATCTCTCCACTGGCTCAGGCTGTGCGGAGCAGCTCTAGAACACCTAGCGATAAGCCTGTGGCTCACGTGGTGGCCAATCCTCAGGCTGAAGGACAGCTGCAGTGGCTGAATAGAAGGGCCAACGCTCTGCTGGCCAACGGCGTGGAACTGAGAGATAATCAGCTGGTGGTGCCCAGCGAGGGCCTGTACCTGATCTATAGCCAGGTGCTGTTCAAAGGCCAGGGCTGCCCTTCTACACACGTGCTGCTGACCCACACCATCAGCAGAATCGCCGTGTCCTACCAGACCAAAGTGAACCTGCTGAGCGCCATCAAGAGCCCCTGTCAGAGAGAAACACCTGAGGGCGCCGAAGCCAAGCCTTGGTACGAACCTATCTATCTCGGCGGCGTGTTCCAGCTCGAGAAGGGCGATAGACTGAGCGCCGAGATCAACAGACCCGACTACCTGGATTTTGCCGAGAGCGGCCAGGTGTACTTCGGCATTATTGCTCTCGGAGGCGGAGGAAGTGGTGGCGGAGGATCTGGCAAGCCCATTCCTAATCCTCTGCTGGGCCTCGACTCCACCTGATGATAA.

AAV-hVEGF—ATGAACTTCCTGCTGAGCTGGGTGCACTGGTCACTGGCTCTGCTGCTGTATCTGCACCACGCCAAATGGTCACAGGCCGCTCCTATGGCTGAAGGCGGAGGACAGAATCACCACGAGGTGGTCAAGTTCATGGACGTGTACCAGCGGAGCTACTGTCACCCCATCGAGACACTGGTGGACATCTTCCAAGAGTACCCCGACGAGATCGAGTACATCTTCAAGCCTAGCTGCGTGCCCCTGATGAGATGCGGCGGCTGTTGTAACGATGAAGGCCTGGAATGCGTGCCCACCGAGGAATCCAACATCACCATGCAGATCATGCGGATCAAGCCCCACCAGGGCCAGCATATCGGCGAGATGTCTTTCCTGCAGCACAACAAGTGCGAGTGCAGACCCAAGAAGGACCGGGCCAGACAAGAGAATCCTTGCGGCCCTTGCAGCGAGCGGAGAAAGCACCTGTTTGTGCAGGACCCTCAGACCTGCAAGTGCTCCTGCAAGAACACCGACAGCAGATGCAAGGCCCGGCAGCTGGAACTGAACGAGAGAACCTGCAGATGCGACAAGCCTAGAAGAGGTGGCGGAGGATCTGGCGGAGGCGGATCTGGAAAGCCCATTCCTAATCCTCTGCTGGGCCTCGACAGCACCTGATGATAA.

### Intravitreal injection

Mice were intravitreally injected with the different AAVs under isoflurane anesthesia and after local anesthetic eye drops were applied (Novesin, OmniVision). Both eyes of 6 mice per group were injected with either 1 × 10^8^ viral genomes (VG)/eye (low dose) or 1 × 10^9^ VG/eye (high dose) in 1 µL AAV buffer. Note that AAV-VEGF was injected at a concentration of 1 × 10^8^ VG/eye and AAV-TNF-α and AAV-IL-6 at 1 × 10^9^ VG/eye with matching AAV-stuffer controls. In an independent mouse cohort, 2 weeks after AAV-TNF-α treatment, eyes were IVT injected with 1 µL of vehicle or neutralizing anti-TNF-α Golimumab (100 mg/mL, Simponi, 4,223,913, Komtur Apotheke). If IVT injection failed (e.g. due to damage of a major blood vessel or the lens), the eye was excluded from analysis.

### In vivo imaging

In vivo imaging was done as described previously^19^. In brief, mice were anesthetized by intraperitoneal injection with 60–90 mg/kg ketamine (Medistar) and 6–8 mg/kg xylazine (Rompun, Bayer). Pupils were dilated with 5 mg/mL tropicamide (Mydrum, Bausch + Lomb) and phenylephrine (Neosynephrin-POS 10%, Ursapharm). A Spectralis HRA/OCT device (Heidelberg Engineering) equipped with a 55° widefield lens was used for recording of Optical Coherence Tomography (OCT) pictures and Autofluorescence (AF) images. For Fundus Fluorescein Angiography (FFA), 200 µL of a 0.2% fluorescein solution (Alcon) were injected subcutaneously and recorded 90 s after injection with the Spectralis HRA/OCT (Heidelberg Engineering). Mice were euthanized by cervical dislocation. The retina and the eye cup (including RPE, choroid, sclera and ciliary body) were dissected, and flash frozen in liquid nitrogen.

### Oxygen induced retinopathy (OIR) model

The OIR experiments were performed according to previously published protocols (Smith et al.^11^). In brief, 7-days-old male and female pups with their mothers were transferred to a hyperoxic chamber (75% oxygen). At P12, oxygen concentration was slowly reduced back to normoxic conditions (21% oxygen) within approximately 3 h. Control mice remained at normoxic conditions for the whole experiment. Mice were euthanized at P12, P13, P14, P15 and P16 by cervical dislocation. Dissected retinae were stored in RNAlater (Invitrogen).

### RNA extraction and quality control (AAV study)

Flash frozen dissected retina and eye cup tissues (n = 72) were homogenized in 700 µL Qiazol (Qiagen) using a Precellys Evolution homogenizer (Bertin Technologies). Retina samples were homogenized by ceramic beads (MPbio, 6913-500) and eye cup samples by metal beads (Bertin Technologies, 15,987,602) and processed in 4 separate batches. Total RNA was extracted using the miRNeasy Micro Kit (Qiagen, 217,084) according to the manufacturer’s tissue protocol including on-column DNase treatment (Qiagen, 79,254). Total RNA samples (36 eye cup, 36 retina) were quantitatively and qualitatively assessed using the fluorescence-based Broad Range Quant-iT RNA Assay Kit (Thermo Fisher Scientific) and the Standard Sensitivity RNA Analysis DNF-471 Kit on a 96-channel Fragment Analyzer (Agilent), respectively. Concentrations averaged at 48.7 ng/µL while RIN ranged from 5.2 to 9.6, with a median at 9.2. Two samples with RIN below 6 were excluded from downstream processing: AAV-stuffer (high) eye cup replicate 4 and AAV-VEGF eye cup replicate 5. See Table 1 for a summary of all samples in the AAV study.

**Table 1:** Summary of all samples sequenced in the AAV study.

Tissue	Treatment	Concentration [VG/eye]	Replicate count
Retina	Non-injected control	–	6
Retina	AAV-stuffer	1 × 10^8^ (low)	6
Retina	AAV-stuffer	1 × 10^9^ (high)	6
Retina	AAV-VEGF	1 × 10^8^ (low)	6
Retina	AAV-TNF-α	1 × 10^9^ (high)	6
Retina	AAV-IL-6	1 × 10^9^ (high)	6
Eye cup	Non-injected control	–	6
Eye cup	AAV-stuffer	1 × 10^8^ (low)	6
Eye cup	AAV-stuffer	1 × 10^9^ (high)	5
Eye cup	AAV-VEGF	1 × 10^8^ (low)	5
Eye cup	AAV-TNF-α	1 × 10^9^ (high)	6
Eye cup	AAV-IL-6	1 × 10^9^ (high)	6

### RNA extraction and quality control (OIR study)

Retinae were dissected from 12 to 16-day postnatal C57BL/6 J mice (n = 32). RNAlater-preserved tissues were disrupted and homogenized in CK14 tubes (VWR, 10144-496) with RLT buffer using a Precellys Evolution homogenizer (Bertin Technologies). Total RNA was then extracted using the MagMAX-96 total RNA isolation kit (Thermo Fisher Scientific, AM1830) including a DNase digestion prior to final elution (Qiagen, 79254). Total RNA samples were quantitatively and qualitatively assessed on a Synergy microplate reader with a Gen5 Take3 module (BioTek), and the Eukaryote total RNA 6000 Nano microfluidic chip (Agilent, 5067-1511) on a 2100 Bioanalyzer system (Agilent), respectively. Concentrations averaged at 136.3 ng/µL while RIN of selected samples were above 8.4. All samples were further processed for library preparation. See Table 2 for a summary of all samples in the OIR study.

**Table 2:** Summary of all samples sequenced in the OIR study.

Tissue	Treatment	Timepoint	Replicate count
Retina	Normoxia (control)	P12	4
Retina	Normoxia (control)	P13	3
Retina	Normoxia (control)	P14	3
Retina	Normoxia (control)	P15	3
Retina	Normoxia (control)	P16	3
Retina	Hyperoxia (OIR)	P12	4
Retina	Hyperoxia (OIR)	P13	3
Retina	Hyperoxia (OIR)	P14	3
Retina	Hyperoxia (OIR)	P15	3
Retina	Hyperoxia (OIR)	P16	3

### RNA sequencing (AAV study)

70 retina- and eye cup-derived RNA samples were normalized on the MicroLab STAR automated liquid platform (Hamilton). Total RNA input of 250 ng was used for library construction with the NEBNext Ultra II Directional RNA Library Prep Kit for Illumina #E7760, together with the NEBNext Poly(A) mRNA Magnetic Isolation Module #E7490 upstream and the NEBNext Multiplex Oligos for Illumina #E7600 downstream (all New England Biolabs). The only deviation from the manufacturer’s protocol was the use of Ampure XP beads (Beckman Coulter) for double-stranded cDNA purification, instead of the recommended SPRIselect Beads. The index PCR was performed with 12 cycles, while the final library was eluted in 35 µL. mRNA libraries were then quantified by the High Sensitivity dsDNA Quanti-iT Assay Kit (ThermoFisher) on a Synergy HTX (BioTek). Library molarity averaged at 149 nM. mRNA libraries were also assessed for size distribution (smear analysis of 360 bp average) and adapter dimer presence (< 0.5%) by the High Sensitivity Small Fragment DNF-477 Kit on a 96-channel Fragment Analyzer (Agilent). All 70 sequencing libraries were then normalized on the MicroLab STAR (Hamilton), pooled and spiked in with PhiX Control v3 (Illumina). The library pool was subsequently clustered on an S4 Flow Cell and sequenced on a NovaSeq 6000 Sequencing System (Illumina) with dual index, paired-end reads at 2 × 100 bp length (Read parameters: Rd1: 101, Rd2: 8, Rd3: 8, Rd4: 101), reaching an average depth of 31.3 million Pass-Filter reads per sample (7.0% CV). The description of RNA library preparation and sequencing closely aligns with the one given in Becker et al.^36^.

### RNA sequencing (OIR study)

Total RNA input of 100 ng was used for library construction with the TruSeq Stranded mRNA LT kit—Set A (Illumina, RS-122-2101), together with the SuperScript II Reverse Transcriptase (Thermo Fisher Scientific, 18064014), according to the manufacturer’s protocol. The quality of mRNA libraries was assessed for adapter and heterodimer presence using the DNA 1000 microfluidic chip (Agilent, 5067-1504), while library molarity was measured using the Quant-iT PicoGreen dsDNA Assay Kit (Invitrogen, P11496). All 32 sequencing libraries were then normalized, pooled and spiked in with PhiX Control v3 (Illumina). The library pool was subsequently clustered using the TruSeq SR Cluster Kit v3 (Illumina, GD-401-3001), and sequenced with TruSeq SBS Kit v3 reagents (Illumina, FC-401-3002) on a HiSeq 2000 Sequencing System (Illumina) with single index, single-read reads at 1 × 51 bp length (Read parameters: Rd1: 52, Rd2: 7), reaching an average depth of 24.9 million Pass-Filter reads per sample (13.1% CV).

### Cell culture

Primary human retinal microvascular endothelial cells (HRMEC, ACBRI 181, Cell Systems Corporation) were cultivated in Endothelial Cell Growth Medium (C-22010, Promocell) on plates pre-coated with 0.1% gelatin (ES-006-B, Millipore). Cells were cultivated in a humidified chamber at 37 °C and 5% CO_2_. The LUNA-FL cell counter (LogosBio) with Acridine Orange dye (F23002, LogosBio) was used for cell quantification before seeding. HRMECs were stimulated with 10 ng/mL recombinant human TNF-α (210-TA-005, RnD Systems) for 24 h at 37 °C. Cells were washed with PBS and lysed in RLT buffer provided by Qiagen and RNA extracted using the RNeasy Mini Kit (74106, Qiagen) according to the manufacturer’s manual.

### qRT-PCR

cDNA synthesis was done using the high-capacity cDNA Kit (4368814, Applied Biosystems) and qRT-PCR was performed with the Taqman Universal PCR Master Mix (4304437, Applied Biosystems) on a QuantStudio 6 Real-Time PCR system (Applied Biosystems). Relative expression (fold induction) was calculated using the ΔΔCT method and the 18S (for mouse tissue) and POLR2A (for HRMECs) genes were used as a normalization controls. The following Taqman probes were used in this study: 18S (Hs99999901_s1), POLR2A (Hs00172187_m1), Madcam1 (Hs00369968_m1), CCL2 (Mm00441242_m1) and a custom-made Taqman probe detecting human codon-optimized VEGF with a V5 tag (fwd primer 5ʹ- ACTGAACGAGAGAACCTGCA -3ʹ, rev primer 5ʹ GCCCAGCAGAGGATTAGGAA-3ʹ, probe 5ʹ- CTAGAAGAGGTGGCG-3ʹ).

### Tissue lysis and ELISA

Eyes for validation of C3 expression were generated from an independent mouse cohort that is published elsewhere^57^. In brief, 2 weeks after IVT injection of AAV-TNF-α, 100 µg golimumab (Simponi) were injected intravitreally. 6 weeks after AAV injection, whole eyes were enucleated, flash frozen in liquid nitrogen, and homogenized in lysis buffer (9803, Cell Signalling) supplemented with proteinase inhibitor Pefabloc SC (76307-100MG, Sigma) with metal beads (15987602, Bertin Technologies) in a Precellys Evolution tissue homogenizer (Bertin Technologies). C3 expression was measured with the Mouse C3 SimpleStep ELISA Kit (ab26388, Abcam) according to the manufacturer’s protocol and measured with a SpectraMax Plus 384 plate reader (Molecular Devices).

### Histology

Eyes for histological analysis were generated in an independent experiment that was published elsewhere^19^. Eyes were enucleated 3–6 weeks after IVT injection and directly fixed in 4% paraformaldehyde (AR1068, Boster) for 48 h at 4 °C. Eyes were dehydrated, incubated in xylol, and infiltrated with paraffin using a tissue processing machine (Tissue-Tek VIP 6, Sakura). Immunohistochemical stainings were performed on 3 µm sections with the Opal Multiplex IHC Kit (Akoya Biosciences) and carried out on the automated Leica Bond platform (Leica Biosystems, Melbourne, Australia). Antigen retrieval was done with the BOND Epitope Retrieval Solution 1 (35608, Leica Biosystems, Melbourne, Australia) at 95 °C and pH 6.0 for 20 min. Polyclonal rabbit anti-CD3 antibody (ab5690, Abcam) was diluted 1:200 and used as a pan-T-cell marker. OPAL polymer anti-rabbit-HRP secondary antibody (ARR1001KT, Akoya Biosciences) and OPAL 570 reagent (FP1488001KT, Akoya Biosciences) were used to develop the fluorescent signal. Nuclei were stained with spectral DAPI (FP1490, Akoya Biosciences) and slides were mounted with ProLong Antifade Mounting Medium (P36961, Invitrogen). Immunofluorescence stainings were imaged using a laser-scanning microscope LSM700 (20 × objective, Carl Zeiss Microscopy GmbH). RNAscope in situ hybridizations for human VEGF, TNF-α and IL-6 were performed externally at ACDBio/Biotechne using the RNAscope 2.5 LSx Red assay. Custom-made probes were designed based on the codon-optimized, V5-tagged human transgenes expressed by each AAV. As technical controls, ACD Positive Control (Mm-Ppib, 313918, ACDBio) and ACD Negative Control (dapB, 312038, ACDBio) probes were used. Epitope retrieval was done for 15 min at 88 °C and proteolysis was performed with Protease III for 15 min at 40 °C at ACDBio. Slides were imaged using an AxioScan.Z1 slide scanner (20 × objective, Carl Zeiss Microscopy GmbH).

### Data processing and analysis

In case of the OIR dataset GRCm38.96 was used as the reference genome. For the AAV study, a custom genome was created by merging codon optimized sequences for hIL6, hTNFa, and hVEGF into the mouse genome. Generation of genomic indices and read alignment was carried out via STAR (version 2.5.2b). Quantification on gene level was performed using FeatureCounts (version 1.5.1) and RSEM (version 1.3.0), while discarding multimapping reads. Quality control metrics were obtained using MultiQC (version 1.0), with information assembled from STAR, picardmetrics (version 0.2.4), and fastQC (version 0.11.5), among other sources.

Genes with a read count value of below 10 across all samples in either the AAV or OIR study were removed from our analysis. We normalized counts expression values using the voom function provided by limma (version 3.44.3). In the AAV study we corrected for an identified batch effect associated with the RNA extraction batch (variable: rna_extraction_batch) using the ComBat function (sva package version 3.40.0). Principal component analysis was performed using the pcaMethods R package (version 1.80.0) selecting for the top 1000 most variable genes.

Differential gene expression was calculated using the lmFit function provided by the limma R package. Significantly changing genes were selected based on a BH-adjusted p-value < 0.05. Pathway information was retrieved using the misgdbr R package (version 7.1.1). Gene set enrichment analysis was performed using the over-representation analysis (ORA) function provided by Clusterprofiler version 4.0.2. Direct comparisons between gene sets derived from the AAV experiment and the OIR study were carried out using a hypergeometric test provided by the stats R package (version 4.1.0). Venn diagrams were plotted using ggvenn (version 0.1.9).

Single cell RNA-Seq datasets were obtained from GSE1352229, GSE130636, and GSE135922. If required, human ensembl IDs were mapped to mouse homolog using the biomaRt R package (2.48.2), with host parameter set to http://feb2021.archive.ensembl.org/. Raw counts were further processed using the Seurat R package (version 4.0.3). Cell specific genes were identified for each individual dataset and cell type via the FindMarkers function. Genes were defined as cell specific if the BH-adjusted p-value < 0.01 and the gene was not identified as specific to any other cell type (unique genes only). As above, the overlap between cell specific marker genes and genesets derived from the AAV or OIR studies were quantified by the ORA function part of clusterProfiler.

## Supplementary Information

Supplementary Figure 1.

Supplementary Table 2.

Supplementary Table 3.

## Data Availability

All data has been deposited in the Gene Expression Omnibus (GEO) database and are available under GSE200191 and GSE200195. Code used for this study is available under https://github.com/bi-compbio/AAV_retinopathy_models.
