# Supplementary material for: Transcriptome analysis of AAV-induced retinopathy models expressing human VEGF, TNF-α, and IL-6 in murine eyes
Source: Sci Rep. 2022 Nov 12;12:19395. doi: 10.1038/s41598-022-23065-4 (PMC9653384; doi:10.1038/s41598-022-23065-4)
Supplement: Supplementary file 1 — Supplementary Figure 1. [file 41598_2022_23065_MOESM1_ESM.pdf]

Supplemental Figures

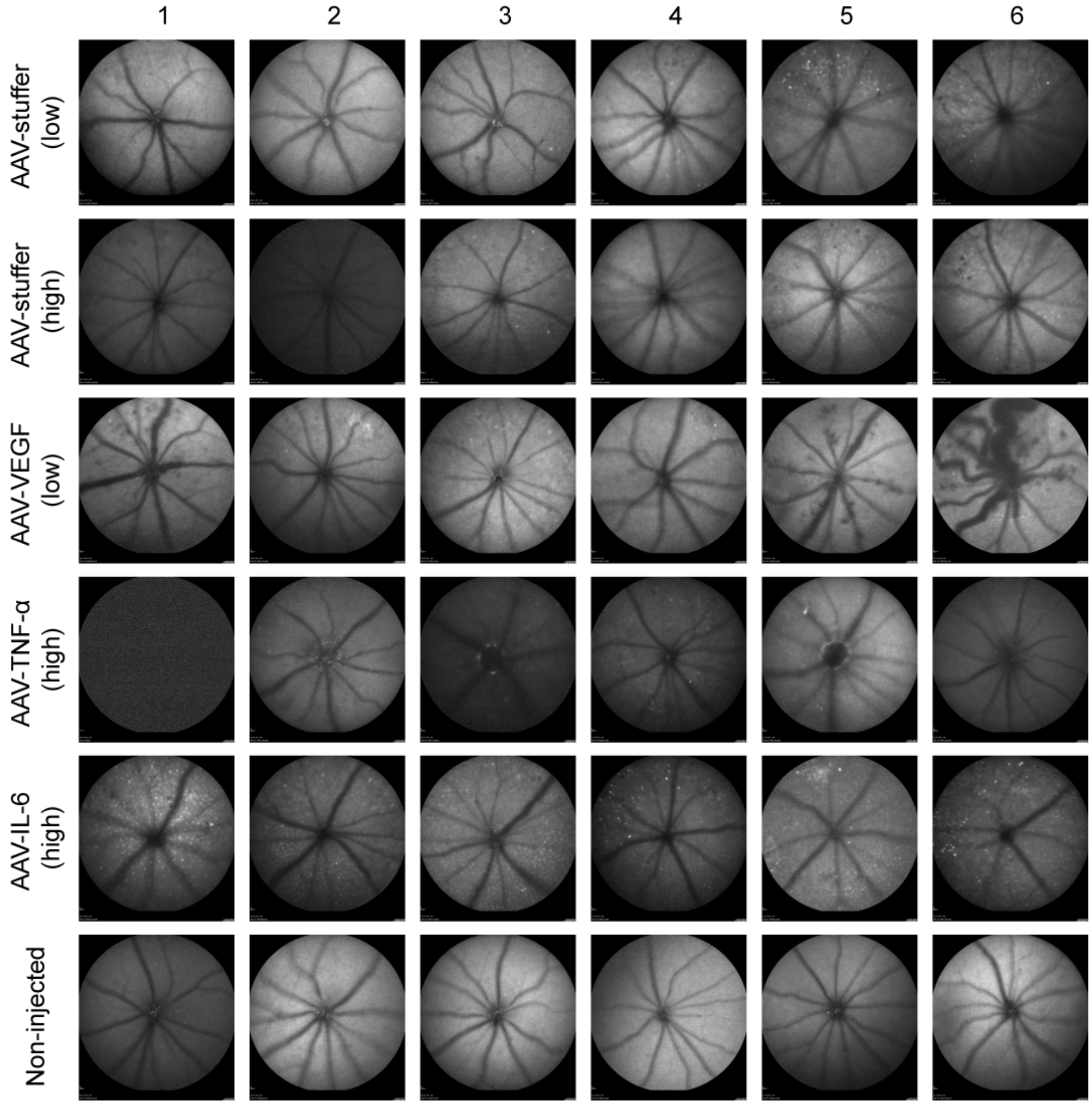

Supplemental Figure 1: Blue autofluorescence fundus (BAF) 3 weeks after IVT injection with AAV-stuffer (control), AAV-VEGF, AAV-TNF-α and AAV-IL-6 and non-injected control eyes. Only AAV-IL-6 injected eyes present hyperfluorescent foci distributed evenly across the eye in the subretinal space. Low dose = 1x108 VG/eye; High dose = 1x109 VG/eye. 6 replicates per treatment.

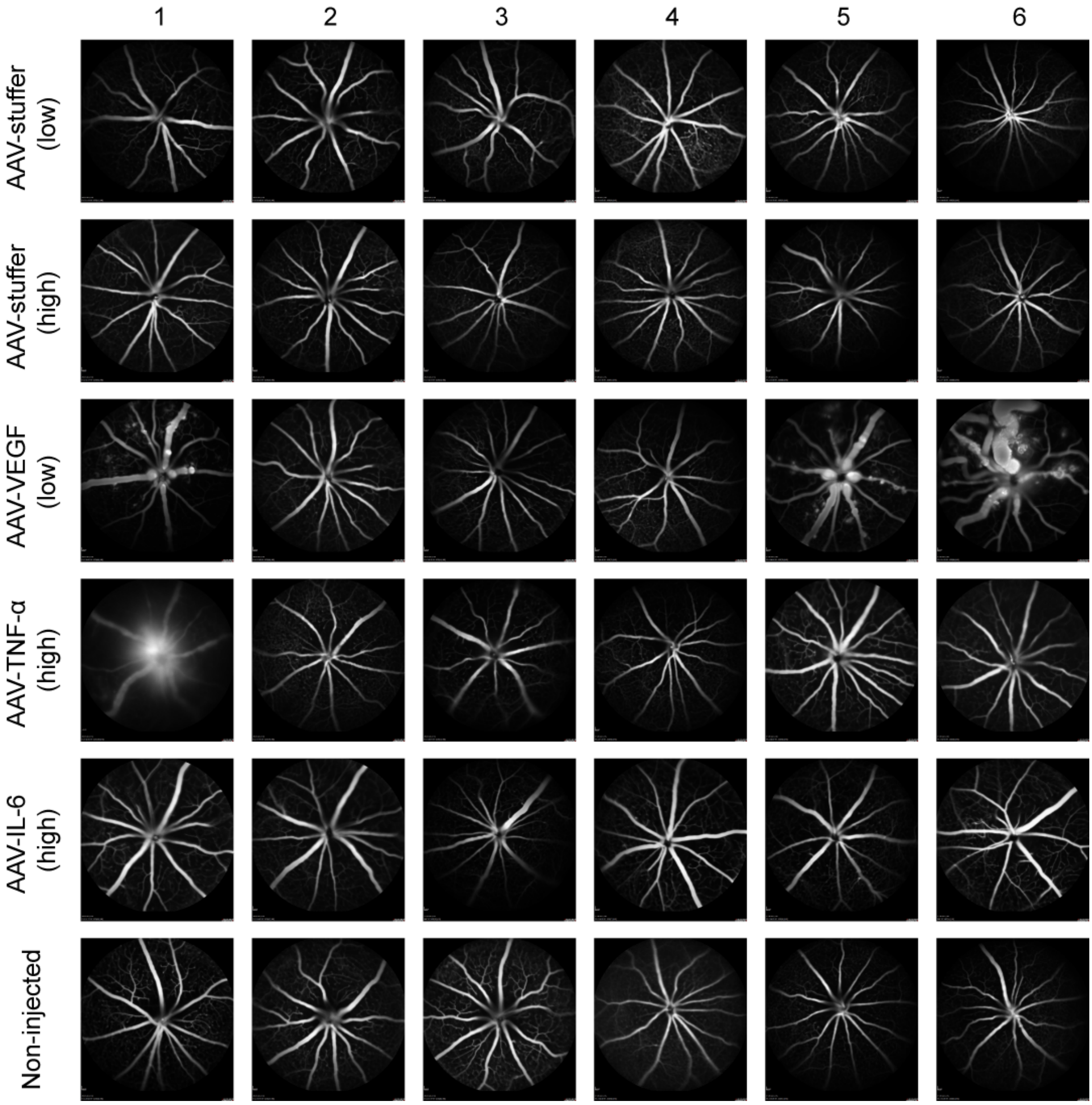

Supplemental Figure 2: Fundus fluorescein angiography (FFA) 3 weeks after IVT injection with AAV-stuffer (control), AAV-VEGF, AAV-TNF-α and AAV-IL-6 and non-injected control eyes. AAV-VEGF injection led to enlarged blood vessels and vascular leakage in 3 of 6 treated eyes. Low dose = 1x108 VG/eye; High dose = 1x109 VG/eye. 6 replicates per treatment.

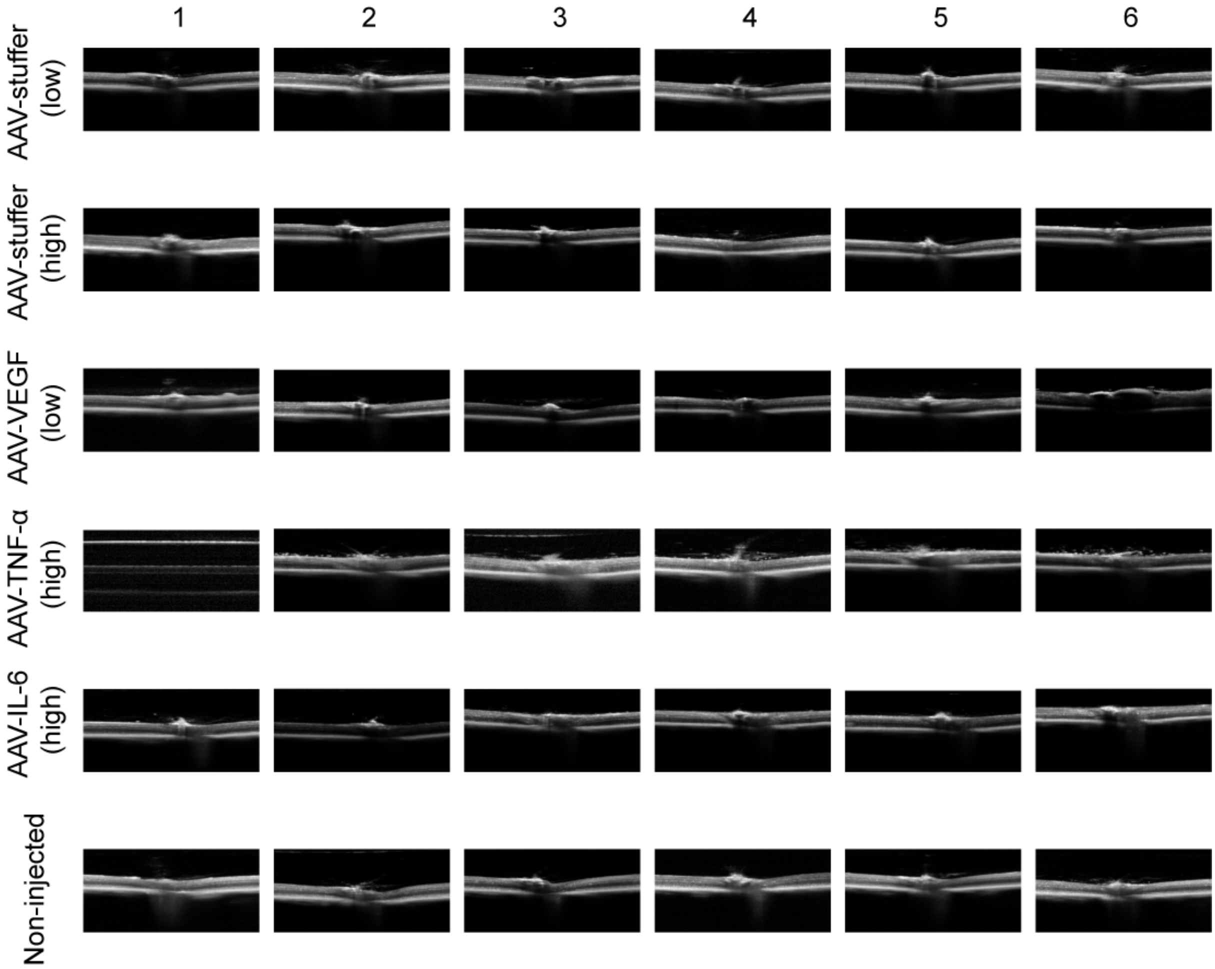

Supplemental Figure 3: Optical coherence tomography (OCT) scans of eyes 3 weeks after IVT injection AAV-stuffer (control), AAV-VEGF, AAV-TNF-α and AAV-IL-6 and non-injected control eyes. Many cellular infiltrates are present in the vitreous of AAV-TNF-α injected eyes. No OCT imaging was possible in the first replicate of AAV-TNF-α due to severe pathologies observed in this eye. Low dose = 1x108 VG/eye; High dose = 1x109 VG/eye. 6 replicates per treatment.

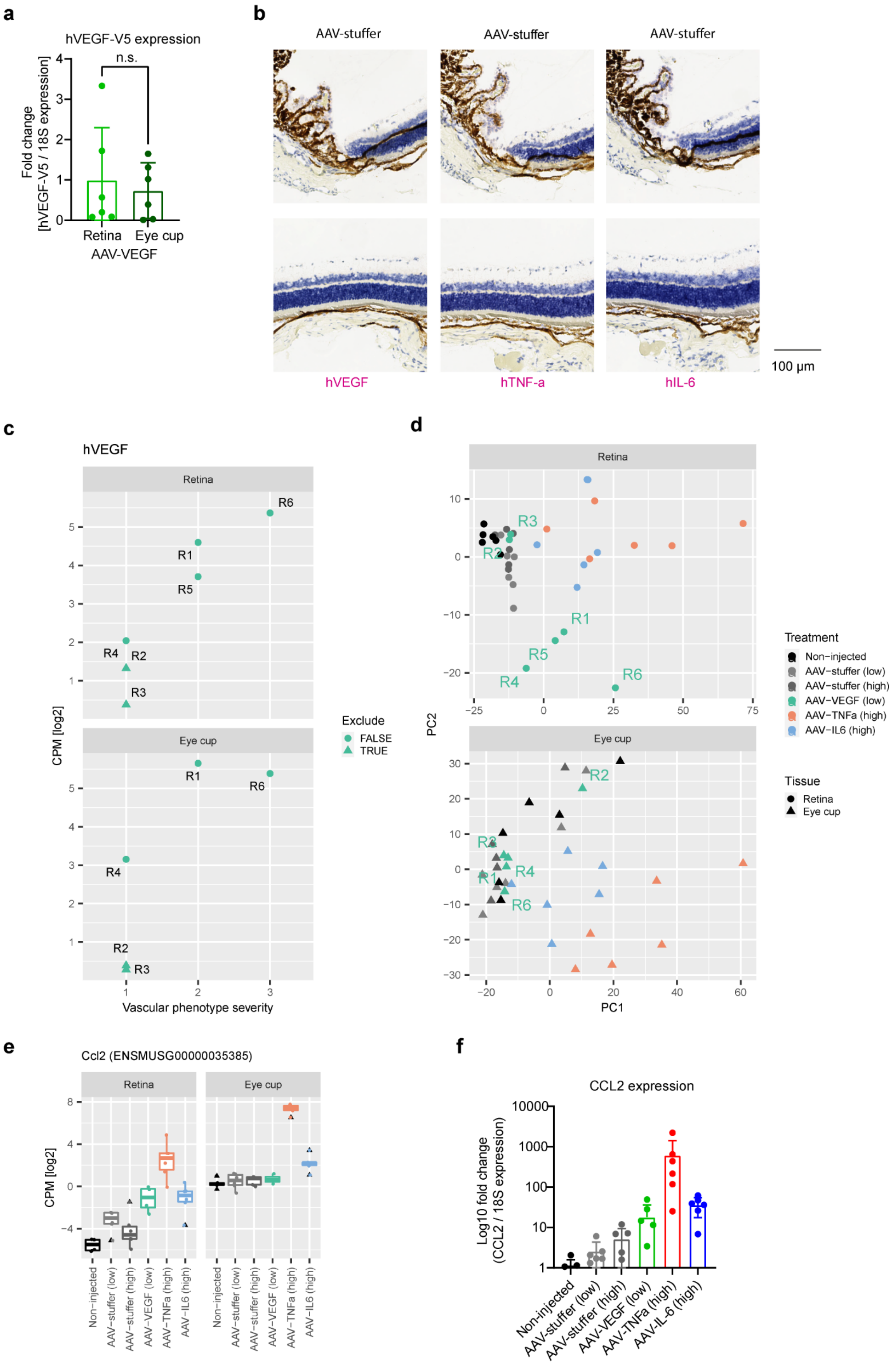
a
hVEGF-V5 expression
n.s.
Fold change [hVEGF-V5 / 18S expression]
Retina
Eye cup
AAV-VEGF
b
AAV-stuffer
AAV-stuffer
AAV-stuffer
hVEGF
hTNF-a
hIL-6
100 μm
c
hVEGF
Retina
Eye cup
CPM [log2]
Vascular phenotype severity
Exclude
FALSE
TRUE
d
Retina
Eye cup
PC2
PC1
Treatment
Non-injected
AAV-stuffer (low)
AAV-stuffer (high)
AAV-VEGF (low)
AAV-TNFa (high)
AAV-IL6 (high)
Tissue
Retina
Eye cup
e
Ccl2 (ENSMUSG00000035385)
Retina
Eye cup
CPM [log2]
Non-injected
AAV-stuffer (low)
AAV-stuffer (high)
AAV-VEGF (low)
AAV-TNFa (high)
AAV-IL6 (high)
f
CCL2 expression
Log10 fold change (CCL2 / 18S expression)
Non-injected
AAV-stuffer (low)
AAV-stuffer (high)
AAV-VEGF (low)
AAV-TNFa (high)
AAV-IL-6 (high)

Supplemental Figure 4: Validation of RNA sequencing results by qRT-PCR and RNAscope® controls (A) qRT-PCR on the same RNA samples used for the RNA seq analysis verified an equal expression of the human VEGF transgene in both retina and eye cup samples (n.s., t-test, n = 6) 3 weeks after IVT injection of AAV-VEGF. (B) Custom-made RNAscope® probes targeting the human transgenes VEGF, TNF-α and IL-6 did not show any staining in AAV-stuffer control eyes. Peripheral retina (upper panel) and the central retina (lower panel). (C) Vascular phenotype severity (based on Supplemental Figure 2) was correlating with the expression level of hVEGF expressed by AAV-VEGF. (D) Replicates 2 and 3 of the AAV-VEGF group clustered together with the controls in the PCA. (E) Expression of CCL2 was highest in AAV-TNF-α injected eyes as determined by RNA sequencing analysis. (F) qRT-PCR on the same RNA samples validated the strong upregulation of CCL2 by AAV-TNF-α.

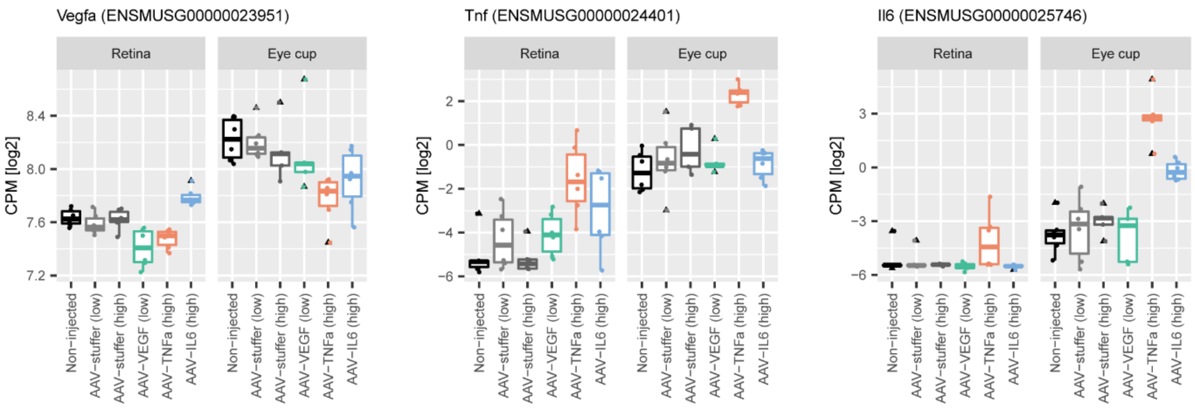

Supplemental Figure 5: Expression of murine endogenous VEGF, TNF-α and IL-6.

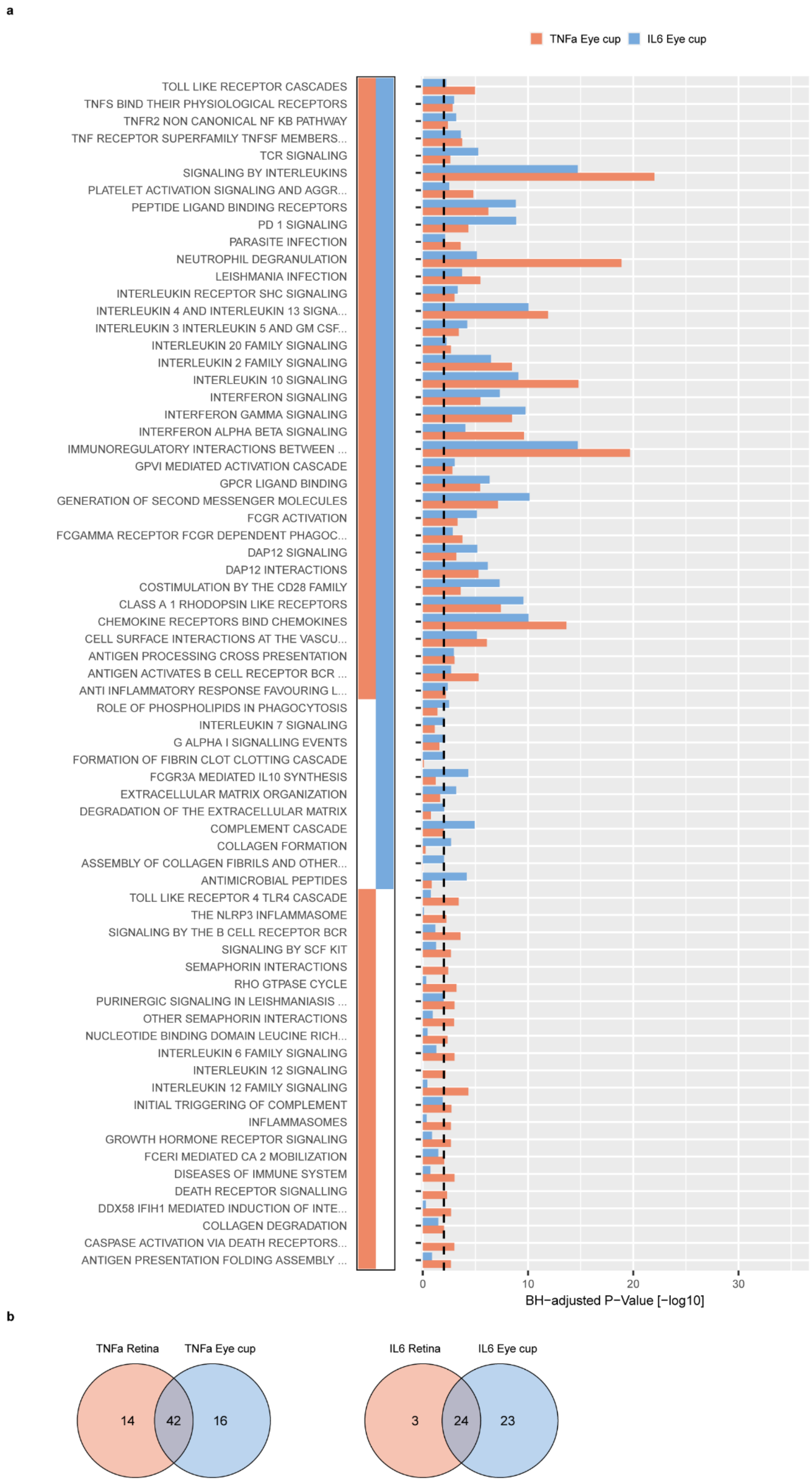
a
TNFa Eye cup
IL6 Eye cup
TOLL LIKE RECEPTOR CASCADES
TNFS BIND THEIR PHYSIOLOGICAL RECEPTORS
TNFR2 NON CANONICAL NF KB PATHWAY
TNF RECEPTOR SUPERFAMILY TNFSF MEMBERS...
TCR SIGNALING
SIGNALING BY INTERLEUKINS
PLATELET ACTIVATION SIGNALING AND AGGR...
PEPTIDE LIGAND BINDING RECEPTORS
PD 1 SIGNALING
PARASITE INFECTION
NEUTROPHIL DEGRANULATION
LEISHMANIA INFECTION
INTERLEUKIN RECEPTOR SHC SIGNALING
INTERLEUKIN 4 AND INTERLEUKIN 13 SIGNA...
INTERLEUKIN 3 INTERLEUKIN 5 AND GM CSF...
INTERLEUKIN 20 FAMILY SIGNALING
INTERLEUKIN 2 FAMILY SIGNALING
INTERLEUKIN 10 SIGNALING
INTERFERON SIGNALING
INTERFERON GAMMA SIGNALING
INTERFERON ALPHA BETA SIGNALING
IMMUNOREGULATORY INTERACTIONS BETWEEN ...
GPVI MEDIATED ACTIVATION CASCADE
GPCR LIGAND BINDING
GENERATION OF SECOND MESSENGER MOLECULES
FCGR ACTIVATION
FCGAMMA RECEPTOR FCGR DEPENDENT PHAGOC...
DAP12 SIGNALING
DAP12 INTERACTIONS
COSTIMULATION BY THE CD28 FAMILY
CLASS A 1 RHODOPSIN LIKE RECEPTORS
CHEMOKINE RECEPTORS BIND CHEMOKINES
CELL SURFACE INTERACTIONS AT THE VASCU...
ANTIGEN PROCESSING CROSS PRESENTATION
ANTIGEN ACTIVATES B CELL RECEPTOR BCR ...
ANTI INFLAMMATORY RESPONSE FAVOURING L...
ROLE OF PHOSPHOLIPIDS IN PHAGOCYTOSIS
INTERLEUKIN 7 SIGNALING
G ALPHA I SIGNALLING EVENTS
FORMATION OF FIBRIN CLOT CLOTTING CASCADE
FCGR3A MEDIATED IL10 SYNTHESIS
EXTRACELLULAR MATRIX ORGANIZATION
DEGRADATION OF THE EXTRACELLULAR MATRIX
COMPLEMENT CASCADE
COLLAGEN FORMATION
ASSEMBLY OF COLLAGEN FIBRILS AND OTHER...
ANTIMICROBIAL PEPTIDES
TOLL LIKE RECEPTOR 4 TLR4 CASCADE
THE NLRP3 INFLAMMASOME
SIGNALING BY THE B CELL RECEPTOR BCR
SIGNALING BY SCF KIT
SEMAPHORIN INTERACTIONS
RHO GTPASE CYCLE
PURINERGIC SIGNALING IN LEISHMANIASIS ...
OTHER SEMAPHORIN INTERACTIONS
NUCLEOTIDE BINDING DOMAIN LEUCINE RICH...
INTERLEUKIN 6 FAMILY SIGNALING
INTERLEUKIN 12 SIGNALING
INTERLEUKIN 12 FAMILY SIGNALING
INITIAL TRIGGERING OF COMPLEMENT
INFLAMMASOMES
GROWTH HORMONE RECEPTOR SIGNALING
FCERI MEDIATED CA 2 MOBILIZATION
DISEASES OF IMMUNE SYSTEM
DEATH RECEPTOR SIGNALLING
DDX58 IFIH1 MEDIATED INDUCTION OF INTE...
COLLAGEN DEGRADATION
CASPASE ACTIVATION VIA DEATH RECEPTORS...
ANTIGEN PRESENTATION FOLDING ASSEMBLY ...
0
10
20
30
BH−adjusted P−Value [−log10]
b
TNFa Retina
TNFa Eye cup
14
42
16
IL6 Retina
IL6 Eye cup
3
24
23

Supplemental Figure 6: (A) Significantly enriched REACTOME pathways in the eye cup tissue of AAV-TNF-α and AAV-IL-6 treated eyes. Both TNF-α and IL-6 mediated expression by AAV led to an enrichment of pathways related to TNF-α signaling and certain interleukins. Only few pathways including fibrin and collagen-related genes were enriched only in AAV-IL-6 injected eyes. AAV-TNF-α injection led to an enrichment of genes in the inflammasome, cell death and specific interleukin related pathways. (B) Venn diagram comparing the number of REACTOME pathways significantly enriched (BH-adjusted p-value < 0.01) in at least one of the treatments (AAV-TNF-α and AAV-IL-6).

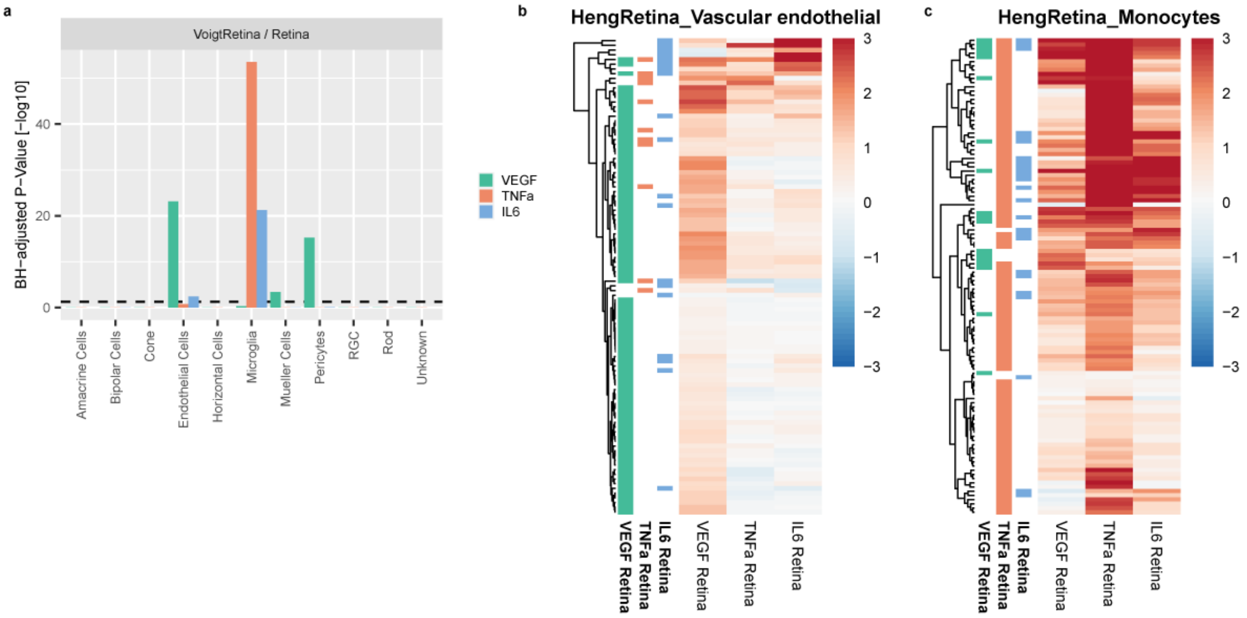

Supplemental Figure 7: Up-regulation of cell type-specific genes. (A) Validation of cell type-specific gene expression with the human scRNA-Seq dataset by Voigt et al. (B) Endothelial cell-specific genes were mostly upregulated in AAV-VEGF treated eyes. (C) Monocyte-specific genes were highly upregulated in AAV-TNF-α treated eyes.

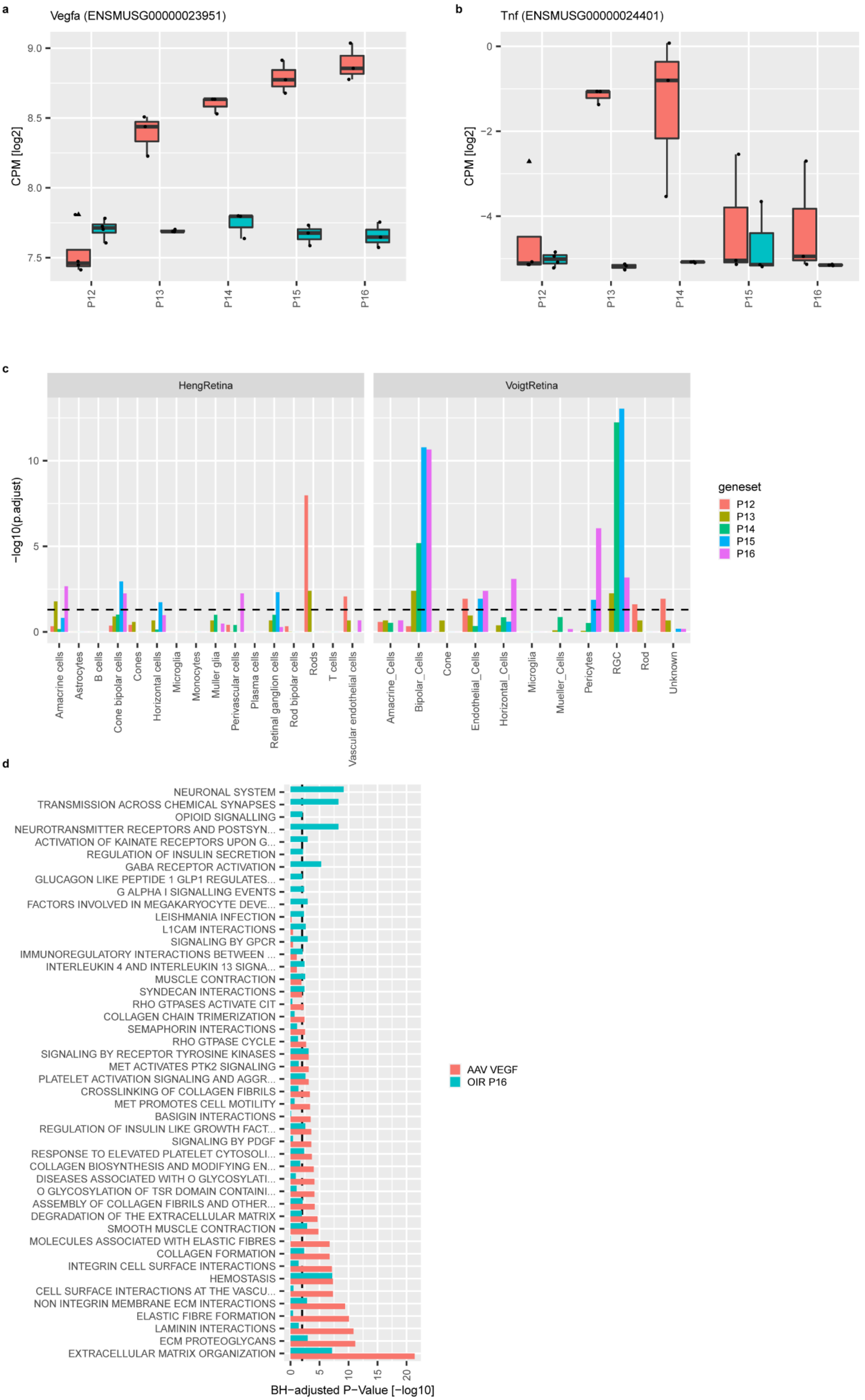

a
Vegfa (ENSMUSG00000023951)
CPM [log2]
9.0
8.5
8.0
7.5
P12
P13
P14
P15
P16
b
Tnf (ENSMUSG00000024401)
CPM [log2]
0
−2
−4
P12
P13
P14
P15
P16
c
HengRetina
VoigtRetina
−log10(p.adjust)
10
5
0
Amacrine cells
Astrocytes
B cells
Cone bipolar cells
Cones
Horizontal cells
Microglia
Monocytes
Muller glia
Perivascular cells
Plasma cells
Retinal ganglion cells
Rod bipolar cells
Rods
T cells
Vascular endothelial cells
Amacrine_Cells
Bipolar_Cells
Cone
Endothelial_Cells
Horizontal_Cells
Microglia
Mueller_Cells
Pericytes
RGC
Rod
Unknown
geneset
P12
P13
P14
P15
P16
d
NEURONAL SYSTEM
TRANSMISSION ACROSS CHEMICAL SYNAPSES
OPIOID SIGNALLING
NEUROTRANSMITTER RECEPTORS AND POSTSYN...
ACTIVATION OF KAINATE RECEPTORS UPON G...
REGULATION OF INSULIN SECRETION
GABA RECEPTOR ACTIVATION
GLUCAGON LIKE PEPTIDE 1 GLP1 REGULATES...
G ALPHA I SIGNALLING EVENTS
FACTORS INVOLVED IN MEGAKARYOCYTE DEVE...
LEISHMANIA INFECTION
L1CAM INTERACTIONS
SIGNALING BY GPCR
IMMUNOREGULATORY INTERACTIONS BETWEEN ...
INTERLEUKIN 4 AND INTERLEUKIN 13 SIGNA...
MUSCLE CONTRACTION
SYNDECAN INTERACTIONS
RHO GTPASES ACTIVATE CIT
COLLAGEN CHAIN TRIMERIZATION
SEMAPHORIN INTERACTIONS
RHO GTPASE CYCLE
SIGNALING BY RECEPTOR TYROSINE KINASES
MET ACTIVATES PTK2 SIGNALING
PLATELET ACTIVATION SIGNALING AND AGGR...
CROSSLINKING OF COLLAGEN FIBRILS
MET PROMOTES CELL MOTILITY
BASIGIN INTERACTIONS
REGULATION OF INSULIN LIKE GROWTH FACT...
SIGNALING BY PDGF
RESPONSE TO ELEVATED PLATELET CYTOSOLI...
COLLAGEN BIOSYNTHESIS AND MODIFYING EN...
DISEASES ASSOCIATED WITH O GLYCOSYLATI...
O GLYCOSYLATION OF TSR DOMAIN CONTAINI...
ASSEMBLY OF COLLAGEN FIBRILS AND OTHER...
DEGRADATION OF THE EXTRACELLULAR MATRIX
SMOOTH MUSCLE CONTRACTION
MOLECULES ASSOCIATED WITH ELASTIC FIBRES
COLLAGEN FORMATION
INTEGRIN CELL SURFACE INTERACTIONS
HEMOSTASIS
CELL SURFACE INTERACTIONS AT THE VASCU...
NON INTEGRIN MEMBRANE ECM INTERACTIONS
ELASTIC FIBRE FORMATION
LAMININ INTERACTIONS
ECM PROTEOGLYCANS
EXTRACELLULAR MATRIX ORGANIZATION
0
5
10
15
20
BH−adjusted P−Value [−log10]
AAV VEGF
OIR P16

Supplemental Figure 8: Expression of VEGF, IL-6 and cell type-specific genes and REACTOME pathway analysis. (A) VEGF expression was upregulated starting from P13 in the OIR treatment group. (B) TNF-α expression was upregulated only at P13 and P14 in OIR animals compared to controls. (C) Cell type-specific genes were significantly enriched in OIR animals at different timepoints. (D) REACTOME pathway enrichment in OIR P16 compared to AAV-VEGF injected retina samples highlighted that only in the OIR model genes related to the neuronal system were regulated. OIR P16 and AAV-VEGF both changed expression of genes classified in ECM- and collagen-related pathways.
